# Incorporation of Organosilicon Motifs in Natural and Synthetic Small Molecules for Anticancer Therapeutics: Current Perspectives and Future Opportunities in Drug Design

**DOI:** 10.3390/molecules31132345

**Published:** 2026-07-03

**Authors:** Rushika Raval, Allyson Yu, Lavernie Chen, Abigail Xinlan Yee, Ruirui Liu, Anna Gribok, Edward Njoo

**Affiliations:** Department of Chemistry, Biochemistry and Physics, Aspiring Scholars Directed Research Program, Fremont, CA 94539, USA

**Keywords:** organosilicon chemistry, silanes, silyl ethers, siloxanes, silicates, medicinal chemistry, natural products

## Abstract

Silicon is among the most abundant elements on Earth, yet its incorporation into organic molecules is atypical in most biological contexts. However, the strategic introduction of silicon, in line with the demonstrated success of the incorporation of other bio-orthogonal elements, has emerged as a powerful approach in medicinal chemistry, enabling access to small molecules with unique chemical, physical, and biological properties that offer improved potency, stability, tolerability, or bioavailability profiles for the discovery and development of anticancer therapeutics. In this review, we describe the direct connection between reactivity and physiochemical paradigms of different classes of organosilicon-containing functional groups and their strategic deployment in small molecule design, including silanes, silyl ethers, siloxanes, and organosilicates. Specifically, we aimed to demonstrate how these strategies can be informed by first principles of reactivity in organosilicon containing functional groups, in both synthetic small molecules and bioactive natural products. Particular emphasis is placed on how silicon replacement and addition can be leveraged beyond simple isosteric carbon replacement, and how consequent structure–activity relationships arising from installation of diverse organosilicon motifs can also serve a unique role in unveiling new aspects of biological mechanism and function. Ultimately, the growing body of literature in applications of organosilicon-containing anticancer small molecules and the increasing sophistication and selectivity of synthetic methods used to construct these motifs will undoubtedly continue to expand the appreciation of organosilicon-based functional groups in the medicinal chemist’s toolbox.

## 1. Introduction

**Figure 1:**
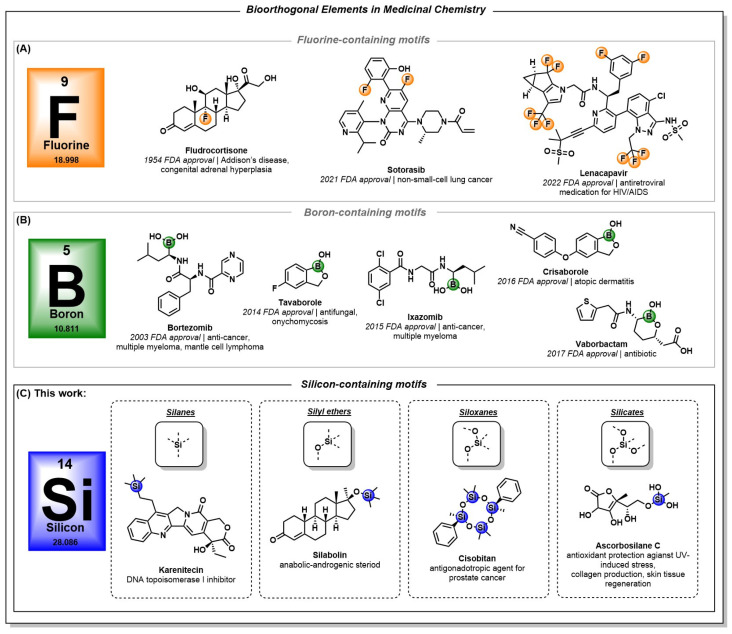
Bioorthogonal elements incorporated in medicinal chemistry and FDA-approved small molecule drugs. (**A**) The incorporation of fluorine in biologically active scaffolds originated from the development of Fludrocortisone in 1954 for the treatment of neurodegenerative diseases, which was followed by Sotorasib and Lenacapavir in 2021 and 2022 respectively. (**B**) The five boron-containing small molecules, Bortezomib, Tavaborole, Ixazomib, Crisaborole, and Vaborbactam, were FDA-approved for the treatment of cancer, multiple myeloma, and dermatitis. (**C**) Silicon-containing functionalities, such as silanes, silyl ethers, siloxanes, and silicates, on complex small molecules have been recently explored to better characterize how this elements’ unique stereoelectronic properties are implicated in biological systems.

The deliberate installation of non-biocanonical elements into druglike molecules allows for the exploitation of unique modalities in chemical interactions, evasion of conventional means of biological metabolism, and augmentation of pharmacological, pharmacodynamic, and pharmacokinetic profiles [1,2,3,4,5,6,7,8]. As a well-precedented example of this, the incorporation of fluorine in biologically active scaffolds traces its origins to the initial development of fludrocortisone and related fluorinated corticosteroids (Figure 1A) [5,9,10,11,12,13,14]. This strategy has since been broadly used to augment biological properties and now accounts for over twenty percent of therapeutics approved by the Food and Drug Administration (FDA) [15]. Analogously, the clinical success of boronic acid-based covalent warheads as protease and proteasome inhibitors unlocks new mechanisms within tactical bioisosterism in the development of small molecule protein inhibitors, including five that have advanced to full FDA approval (Figure 1B) [6,7,16,17,18]. Together, these precedents establish a general strategy in which non-native elements are deployed to access orthogonal chemical space, providing a clear rationale for extending this approach to silicon-based motifs. Historically, organosilicon-containing functional groups have been extensively deployed in synthetic campaigns as protecting groups, reactive intermediates, and diversifiable building blocks [19,20]. More recently, these silicon-containing functionalities have been an emergent arena of exploration in both natural product derivatization and in de novo small molecule design, especially in connection to the development of anticancer therapeutics [1,2,3,21,22,23,24] (Figure 1C).

The applications of organosilicon functional groups include a range of reactivity paradigms of the silicon atom governed by their stereoelectronic properties [25]. These unique characteristics of silicon-containing motifs are not only reflected in their different use cases, synthetic installation, and fundamental reactivities, but also in how these functionalities behave in biological systems [26,27,28]. Silanes, being quaternarily bonded to carbon, bear the greatest electron density, and thus have been synthetically employed as masked carbon nucleophiles. In many biological applications, this rich electron density imbues unique lipophilicity for drug-like molecules with a diverse set of applications, from improving binding through targeted engagement of hydrophobic biomolecular pockets or through electron-donating modulation of biologically active aromatic systems. Beyond this, difunctionalized organosilanes have found a unique set of applications as isosteric spacers that have torsional flexibilities and lipophilicity profiles distinct from analogous hydrocarbon counterparts.

Likewise, silyl ethers, which bear a single silicon–oxygen bond, are among the most common synthetic hydroxyl protecting groups, whose hydrolytic stability and electronic properties can be tailored by their alkyl substituents [29,30,31,32,33]. This direct tunability of hydroxyfunctionalization has been made eminent in the exquisite selectivities of deprotection and protection sequences that have enabled the total synthesis of many complex natural products [33,34,35]. Translated into biological contexts, the versatility of different silyl ether groups range in application from being hydrolytically labile prodrugs to being used as metabolically stable protected hydroxyl groups. In the realm of biologically active natural products, the chemoselective deployment of diversely functionalized silyl ethers offers new opportunities to explore structure–activity relationships.

Given their chemical stability, siloxanes, which bear two silicon–oxygen bonds, have been extensively used not only in modern materials, but also in biopolymers and in drug delivery applications [36,37]. Cisobitan [38], one of the few siloxane-containing small molecules that have advanced to preclinical and clinical evaluation for the treatment of ER-dependent cancers, demonstrates a reapplication of cyclic siloxanes as a core scaffold in the synthetic estrogens space with remarkable chemical and metabolic stability. While these motifs are extensively used in the industrial production of inert polymers and in modern materials, their deployment in small molecules has been more sparse.

Silicates, which bear three to four Si-O bonds, are electrophilic silicon sources, which are known to undergo spontaneous hydrolysis in biological contexts [39]. Taking advantage of this reactivity, current efforts have re-applied this functional group as a prodrug strategy or in the production of intentionally hydrolyzable biopolymeric materials. The tunable hydrolytic stability of alkylsilicates is exemplified in a recent set of examples involving silicate prodrugs of paclitaxel and other anticancer small molecules. Separately, others have shown that the introduction of caged dative bonds can reverse the reactivity of otherwise electrophilic silicates.

**Figure 2:**
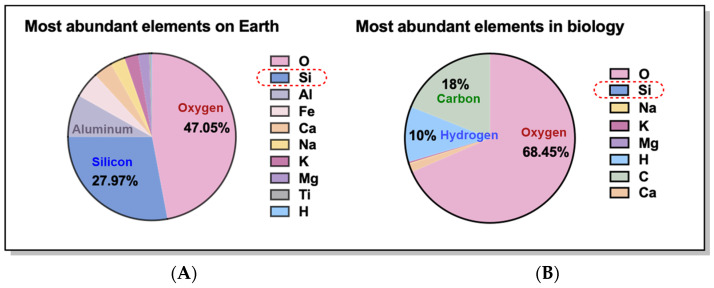
Comparison of elemental abundances on Earth versus those commonly found in living systems. (**A**) Silicon is the second most abundant element on earth, accounting for 22 percent by mass. (**B**) Silicon-containing compounds are virtually nonexistent in naturally occurring biological molecules.

Notwithstanding its status as the second most abundant elements on Earth, the incorporation of silicon into biological molecules is not typically found in naturally occurring biomolecules (Figure 2), presenting a unique opportunity for medicinal chemists and natural product chemists to introduce new means and strategies of small molecule design. In this review, we systematically examine the diversity of the strategic installation of this range of sila-functionalities in anticancer small molecules, with a distinct focus on the impact of such modifications on the biological activity of natural products and their analogs. We compiled a selection of the primary literature of silicon-containing small molecules with reported anticancer activity, ranging from the first examples of organosilicon incorporation in a therapeutic lead (Cisobitan, 1983 [38]) to contemporary examples in the present day (2026). Silicon-containing compounds in the peer reviewed and patent literature with known biological potency in cancer models were identified from substructure and structure search queries in the CAS BioFinder and CAS SciFinder databases [40,41,42] to identify small molecules with specific classes of silyl functional groups with known biological data, which supplemented the contributions from our laboratory and from others to this space. The results from this search were further constrained to biological applications that are mechanistically pertinent to cancers, which is the focus of this present review. We specifically highlighted studies on small molecule structure and function spanning the last five decades that demonstrate the direct translatability of fundamental reactivity paradigms of silicon-containing functional groups on subsequent biological function. While several have undertaken the systematic documentation of bioactive silicon-containing compounds known in the literature, in this review we assess a selection of seminal studies from the perspective of the role of physical organic chemistry reactivity principles. Of note, several examples surveyed here demonstrate the direct effect of carbon to silicon single-atom replacement as a means to alter the original biological function of small molecules. We further explore the recapitulation of known silicon reactivity paradigms to achieve biological profiles unique to bioorthogonal silicon incorporation.

## 2. Alkylsilanes

Alkylsilanes exhibit distinct stereoelectronic properties that differentiate them from their carbon analogs and can be leveraged to modulate the physicochemical behavior of small molecules, offering alternative means of engaging hydrophobic interactions between these drugs and their biochemical targets. Owing to the low electronegativity of silicon and high polarizability of the carbon–silicon bond, alkylsilanes impart greater electron density and lipophilicity compared to carbon-carbon bonds. Sterically, C-Si bonds are moderately longer than their analogous C-C counterparts (1.85 Å vs. 1.54 Å) [2,21] while silicon offers a slightly larger van der Waals radius (210 pm) over carbon (170 pm). Interestingly, the rotational barrier of a tertiary silicon atom is significantly lower than its carbon-containing counterpart, largely as a function of the longer Si-C bond (1.4 kcal/mol vs. 4.0–4.6 kcal/mol in tetramethylsilane vs. *tert*-pentane as model systems) [43,44,45]. These minor physical differences in chemical structure, summarized in Table 1, bear significant consequences on the manner in which alkylsilanes interact with biological targets; a simple carbon to silicon switch can alter binding interactions within hydrophobic pockets, potentially improving biochemical recognition, or access to regions of space that are less available to purely hydrocarbon frameworks. In addition, the increased polarizability of silicon may strengthen dispersion interactions, while the longer bond lengths can subtly expand molecular dimensions without introducing additional functional groups. Together, these effects provide a rationale for the incorporation of alkylsilanes as bioisosteric replacements in medicinal chemistry, where they can be used to fine-tune potency, selectivity, and pharmacokinetic properties.

### 2.1. Synthesis of Alkyl and Arylsilanes

**Figure 3:**
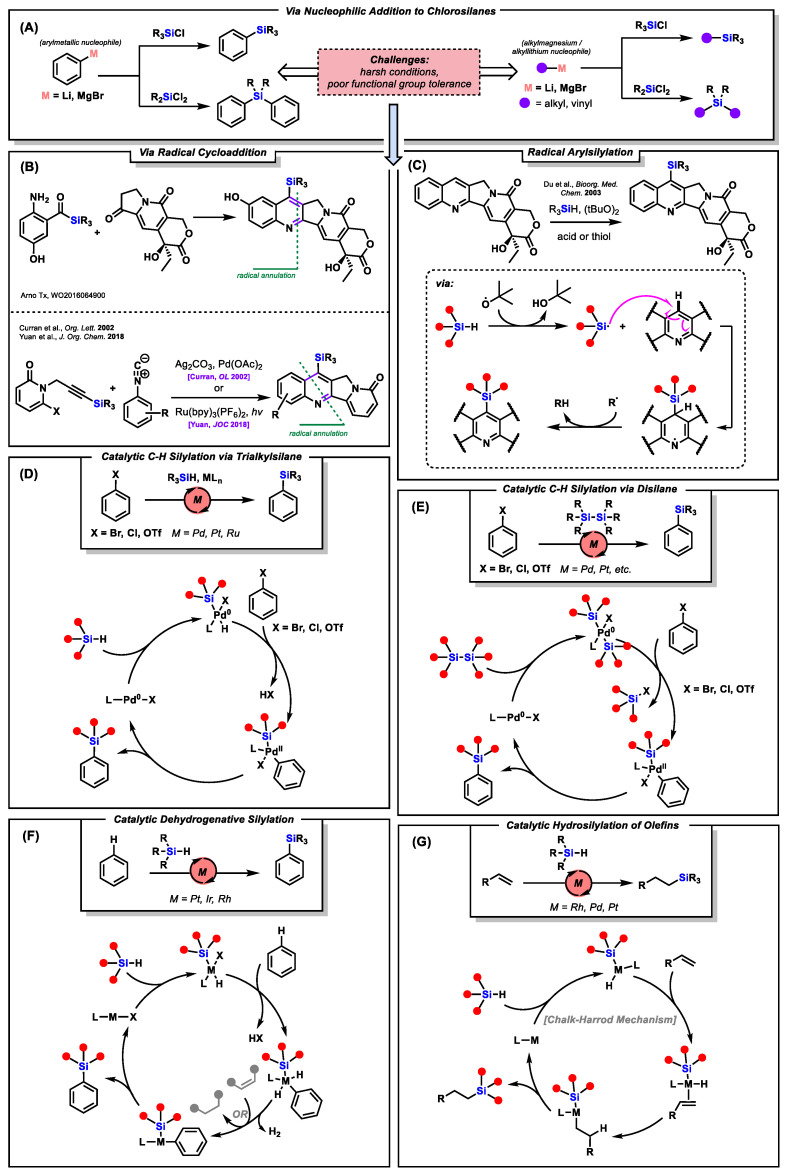
Strategies in synthetic preparation of aryl and alkylsilanes. (**A**) Nucleophilic additions of carbon-centered nucleophiles onto chlorosilanes present one of the simplest methods to construct C-Si bonds in the formation of alkyl and arylsilanes, but are limited in subtrate scope [50,51]. (**B**) Representative radical cascade annulation strategies to access the camptothecin core architecture [52,53,55]. (**C**) Recent acid- or thiol-mediated C-H silylation of camptothecins reported by the Curran group [56]. (**D**) Representative catalytic cycle of aryl silylation of aryl halides via metal-mediated C-X insertion. (**E**) Representative catalytic cycle of installation of arylsilanes via disilanes. (**F**) Representative catalytic cycle for metal-catalyzed dehydrogenative aryl silylation from aryl C-H bonds. (**G**) Mechanistic catalytic cycle for the Chalk-Harrod mechanism for metal-catalyzed hydrosilylation of olefins.

Early preparation of arylsilanes and alkylsilanes relied on organometallic nucleophilic additions to chlorosilane electrophiles [50,51]. The synthesis of arylsilanes and alkylsilanes from commercially available silyl building blocks ranges from direct reaction of nucleophiles with silyl chlorides to catalytic approaches in constructing C-Si bonds with emerging advances in organometallic catalysis. Several examples of biarylsilanes and arylsilanes, for example, can be readily prepared from the nucleophilic addition of arylmagnesium Grignards or aryllithium nucleophiles to dialkyldichlorosilanes, or trialkylcholorosilanes, respectively (Figure 3A). While directly accessible from commercially available and relatively inexpensive dialkyldichlorosilanes or trialkylchlorosilanes, respectively, these conditions are not tolerated on scaffolds with more sensitive functionalities. Other approaches for the construction of arylsilanes involve radical cascades of silylated building blocks; this strategy has been deployed in several examples in the preparation of silane analogs of camptothecin, a plant-derived topoisomerase poison. The early work of Arno Therapeutics [52] demonstrates a highly convergent strategy in constructing the camptothecin B-ring via a radical cascade of a 2-amino arylacylsilane and a densely functionalized C/D/E-ring pyrrolidone fragment, indicating a high degree of functional group tolerance for this endgame cyclization. A later work by the Curran laboratory demonstrated that a different, 4 + 1 radical annulation cascade could be leveraged using a silylated variant of an N-propargylhydroxypyridine and an aryl isocyanide with catalytic palladium (II) acetate (Figure 3B) [53,54]. Following this early example, Yuan and coworkers demonstrated several years later that this same radical annulation could be performed photochemically with a Ru(bpy)_3_(PF_6_)_2_ photoredox catalyst [55]. These strategies in preparing aryl silanes work well in the late-stage construction of silylated arylpyridine motifs such as those found in camptothecin, but do not generally allow for late stage silylderivatization of other ring systems. The Curran laboratory subsequently reported a direct, acid- or thiol-mediated radical silylation of the camptothecin skeleton directly from treatment of the quinoline ring with trialkylsilane and a tert-butylperoxide radical oxidant (Figure 3C) [56]. Inspired by advances in metal-catalyzed borylation catalysis of aryl rings, and in efforts to generalize this ability to functionalize aryl rings catalytically, the Hartwig group and others [57] have demonstrated several examples of Pd^0^/Pd^II^-catalyzed installation of aryltrialkylsilanes from aryl halides, which proceeds through initial oxidative addition of the Pd catalyze across the C-X bond, followed by metal-mediated Si-H bond activation, and final reductive elimination of the aryl-silane bond and regeneration of the metal catalyst (Figure 3D) [58,59,60,61]. Analogous to the mechanistic advances made in recent borylation chemistry, similar reactivity has been achieved with disilanes (e.g., hexamethyldisilane) as a trialkylsilane source [62,63,64] (Figure 3E). Most recently, direct, dehydrogenative installation of silanes on unactivated aryl C-H bonds has been demonstrated in the literature [65,66,67,68,69,70,71], though regio- and chemo-selective aryl C-H silylation remains largely dependent on auxiliaries and directing groups (Figure 3F). Analogous to this, hydrosilylation through a Chalk–Harrod mechanism and related catalytic cycles [72,73,74] remains a longstanding and dependable means to construct C(sp^3^)-Si bonds through initial oxidative addition of a metal catalyst into the Si-H bond of a silane, followed by the activation of an alkene or analogous system from the metal silahydride intermediate. Subsequent β-hydride insertion followed by reductive elimination forges the terminal C-Si bond, thus functionalizing an alkene, alkyne, or other synthon into an alkylsilane (Figure 3G). Though this and similar reactions have been known for decades, new advances in catalyst design and synthetic methodology have enabled milder, more selective, and more efficient hydrosilylation transformations. We anticipate that these and other advances in new methods to forge aryl- and alkylsilanes will enable broader access to silylated bioactive compounds.

### 2.2. Silaplatin: Si Exchange on a Pt(II) DNA Intercalator

The history of silane containing therapeutics began with a remarkably straightforward modification: replacing a single carbon atom with silicon to improve metabolic stability without sacrificing the molecule’s original efficacy. Since its discovery in 1845 [75,76], cisplatin (PtCl_2_(NH_3_)_2_) (Compound **4.1**) has inspired a family of Pt-based DNA replication inhibitors [77,78,79] that function through cross-linking of DNA base pairs. Cisplatin putatively engages DNA cross linking through initial hydration of the aqua monochlorodiamine complex in chloride-depleted intracellular environments, and this resulting Lewis acidic Pt species undergoes transmetallation of the chloride and water ligands with guanine and other Lewis basic nucleotides, ultimately triggering DNA-repair mechanisms that elicit apoptotic death in rapidly dividing cells. The potency and rate of Pt-induced cell death is well reported to be a function of the kinetics of ligand exchange and dichloride hydrolysis, which can be, in turn, tuned by ligand stereoelectronic effects [80].

Early optimization of cisplatin has led to the development of cyclic diamine ligand-based systems, which are known to entropically and electronically stabilize an otherwise very Lewis acidic platinum (II) species, leading to the discovery of carboplatin [81,82], oxaliplatin, and others [83,84]. Substitution of carbon with silicon within the diamine framework introduced additional electronic effects. In particular, β-silyl substitution is known to increase the basicity of adjacent amine functionalities, as reflected in the higher pK_a_ of the corresponding siladiamine relative to its carbon analog. Incorporation of such ligands into Pt(II) complexes can, therefore, alter the donor strength of the amine and, consequently, the electron density at the metal center, with downstream effects on aquation kinetics and DNA-binding reactivity.

**Figure 4:**
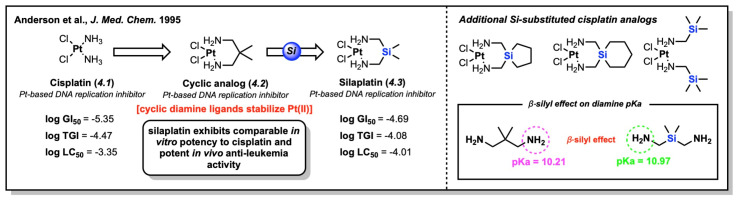
Silaplatin as a first example of silane-containing anticancer drugs [85]. Cisplatin, a Pt(II) complex initially developed in the 19th century for the treatment of cancers, functions through initial ligand exchange of the chlorides followed by DNA binding and eventual cross-linking of DNA. Pt(II) complexes prepared with cyclic diamine ligands have demonstrated improved hydrolytic stabilities and/or bioavailability profiles, making this an attractive strategy to tune the reactivity of cisplatin derivatives. Taking advantage of the minor changes in pK_a_ and Lewis basicity from the β-silyl effect, Anderson and coworkers prepared the first silane and spirosilane-containing cisplatin derivatives, including silaplatin (**4.3**) toward the development of more potent cisplatin analogs with comparable biological potency.

Inspired by the essential role that ligand sterics and electronics play in fine-tuning the reactivity of the platinum electrophile, Anderson and coworkers designed, prepared, and evaluated a library of carbocyclic and silacyclic diamine complexes of Pt(II) as lead compounds for the treatment of a broad spectrum of cancers, including the L1210 leukemia line in murine models [85]. Expectedly, a cyclic Pt(II) complex bearing a 2,2-dimethyl-1,3-diamine ligand **4.2** exhibited improved hydrolytic stability (t_1/2_ = 238 min.) as compared to its acyclic analog. By leveraging a β-silyl effect [86] on the relatively higher pKa of an analogous siladiamine (pK_a_ = 10.97 vs. 10.21), Anderson and coworkers prepared a dimethylsiladiamine complex **4.3** along with several cyclic spirosilanes and acyclic trimethylsilylmethylamine (Figure 4). While the cyclic silyl complexes exhibited comparable hydrolytic stabilities to cisplatin, limited changes were observed in overall anticancer potency.

In an NCI panel of cancers, silaplatin exhibited comparable antitumor efficacy as cisplatin, and demonstrated more durable potency in vivo against cisplatin-resistant cell lines, particularly in murine leukemia models. These findings suggest that incorporation of silicon into ligand frameworks provides a viable strategy for modulating the reactivity and biological performance of Pt(II) complexes.

### 2.3. ATX020: A Silapiperidine for KIF18A Inhibition

**Figure 5:**
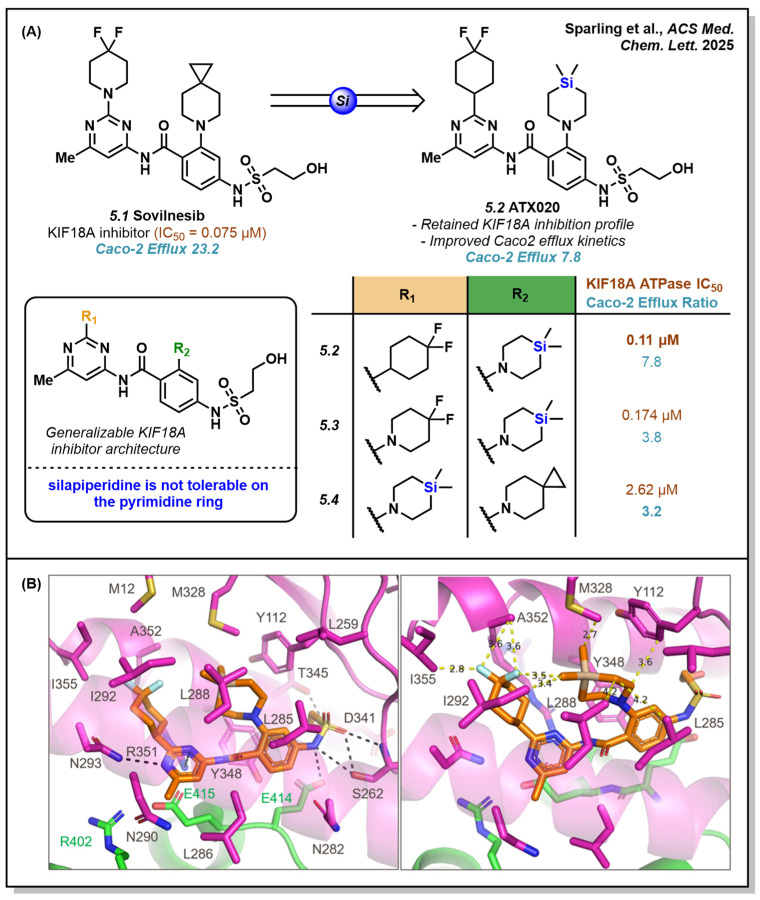
Incorporation of a silapiperidine on KIF18A inhibitors improves PGP efflux dynamics. (**A**) Structural analogy of previously-reported KIF18A inhibitor Sovilnesilib **5.1** and ATX020 **5.2**, which differs in the replacement of a B-ring cyclopropylpiperidine motif with the corresponding silapiperidine evades PGP efflux while retaining potency; alternate replacement of an A-ring gem-difluoropiperidine lowers PGP efflux at the expense of KIF18A ATPase potency. (**B**) Docked structure of ATX020 bound to the ATPase pocket of KIF18A demonstrates productive interaction of the B-ring silapiperidine with hydrophobic residues I292, Y112, L288, and M328 (Docking structures reproduced with permission from Sparling, et al. *ACS Med. Chem. Lett.* **2025** ([87]), © American Chemical Society, 2025).

A recent manifestation of this carbon-to-silicon replacement strategy was deployed in the development of ATX020 (Compound **5.2**), a highly potent, selective, and efflux-resistant KIF18A inhibitor reported by Sparling and team from Accent Pharmaceuticals [87]. The KIF18A kinase, a key regulator of tubulin polymerization, has been an attractive target for the development of antiproliferative small molecules given the mechanistic ubiquity of antimitotic agents [88,89] and the unique sensitivity of chromosomally unstable cancer cells to KIF18A inhibition [90,91,92]. This selectivity on TP-53 loss-of-function cancer cells attenuates risk of off-target antiproliferative activity in normal cells [87,90,91,92], and has previously led Tamayo and team from Amgen towards the development of Sovilnesib (Figure 5A, Compound **5.1**), which uniquely bears two piperidine rings that, in close spatial proximity, occupy a hydrophobic pocket in an allosteric KIF18A binding region [91] (Figure 5B). This initial lead compound, while exhibiting highly potent in vitro activity against KIF18A (IC_50_ = 75 nM), suffered from a known drug efflux liability due to its promiscuity as a PGP substrate. Recognizing that this pharmacokinetic deficiency may be ameliorated with structural tailoring of specific hydrophobic elements in this scaffold, Sparling et al. demonstrated that substitution of either the 4,4-difluoropiperidine ring on the pyrimidine fragment or the 4-cyclopropylpiperidine ring on the benzamide fragment with a dimethylsilapiperidine generally leads to a decrease in PGP-mediated efflux by nearly an order of magnitude. This discovery validated an earlier mechanistic hypothesis that hydrophobicity tuning of the aliphatic groups with a silacycle would diminish the propensity of such scaffolds to be effluxed by PGP. Remarkably, while both ATX020, which bears a dimethylsilapiperidine in place of the 4-cyclopropylpiperidine fragment, and its closely related congener **5.4**, exhibit desirable improvements in Caco-2 permeability, the latter revealed a precipitous loss of KIF18A potency (IC_50_ = 2.62 μM) compared to ATX020 (IC_50_ = 0.11 μM). This may be attributed to the relative spatial demand of the two piperidine rings within the KIF18A binding pocket as well as a loss of solubility incurred by replacement of the fluorinated piperidine ring with the diemethylsilapiperidine equivalent.

The uniform improvements in PGP efflux liability demonstrate that silane-based bioisosterism offers a generalizable strategy for improving drug permeability on these scaffolds. Simultaneously, the substantial difference in endgame potency, observed in these two highly homologous dimethylsilapiperidine analogs of sovilnesib indicate that the success of this bioisosteric replacement is dependent on site-specific target contextualization.

### 2.4. Silylated Camptothecins: DB-67 and Karenitecin

Antimitotic agents represent the largest mechanistic class of plant derived allelochemicals, and include several biosynthesized poisons that target the biochemical basis for DNA replication [93]. Given the central role of cell cycle dysregulation as a hallmark of all cancers, targeting these same central mechanisms of DNA replication are an attractive chemotherapeutic strategy for small molecule design, several of which draw inspiration from originally discovered phytochemical scaffolds. Among the most potent of such poisons is Camptothecin (**6.1**), an alkaloid first isolated from the tree *Camptotheca acuminata*, that has been shown to poison topoisomerase I by stabilizing its ternary complex with DNA, preventing re-ligation of the nicked DNA strand in cells, and eventually leading to apoptotic cell death [94,95,96,97]. Although camptothecin (**6.1**) showed potent antitumor activity in preclinical trials, these efforts were terminated due to the compound’s poor solubility (0.01 mg/mL), instability, and off-target toxicity. Consequently, establishing derivatives bearing more favorable solubility and selectivity profiles has been a critical focus towards the development of camptothecin-based anticancer pharmaceutics. Structural functionalization and prodrug strategies at the A/B ring system led to enhanced pharmacological properties, yielding FDA approved drugs topotecan and irinotecan for the treatment of small cell lung cancer, ovarian cancer, and colorectal cancer, respectively. Despite their clinical success as first and second in line therapies for metastatic cancers, a fundamental limitation of camptothecin-based therapeutics arises from the hydrolytic instability of the E-ring lactone pharmacophore upon extended hydrolytic exposure in aqueous medium. While stable at pH < 5.5, the lactone undergoes rapid hydrolysis at physiological pH to form an inactive carboxylate, which binds with significantly higher affinity than its parent compound to human-serum albumin [98,99]. While some derivatives bearing substitutions at the C7/C9/C10 position have demonstrated a reduction of this preferential binding, experimentation in whole blood systems in vitro have demonstrated topotecan only retains 11.9% of its native lactone, irinotecan with 21.0%, and SN-38 with 19.5% at equilibrium. Thus, the establishment of rational structural modifications favoring increased membrane permeability and intracellular drug exposure would be a modality towards improving the therapeutic efficacy of camptothecin derivatives.

Among these strategies, the introduction of organosilicon containing side chains has emerged as a particularly compelling solution. The first generation of silane modified camptothecins were enabled by a modular cascade radical annulation involving a silyl substituted alkyne and a phenyl isonitrile that led to the initial development of A/B ring functionalized derivatives bearing silyl substituents at C7 on camptothecin and similar scaffolds, where these synthesized derivatives demonstrated high antitumor cytotoxicity [100], providing a streamlined route to access a wide assortment of new CPT derivatives. One such example is DB-67 (**6.4**), bearing a *tert*-butyldimethylsilane at C7 and hydroxylation at C-10 developed by Bom and coworkers. This compound, devised to enhance blood solubility while retaining potency and lipophilicity, was then assessed in non-small cell lung cancer, ovarian cancer, breast cancer, melanoma and displayed dose-dependent antiproliferative activity across all cell lines with increased potency (GI_50_ = 21 nM) comparing to camptothecin (GI_50_ = 44 nM) and SN-38 (GI_50_ = 36 nM). Due to the improved solubility and pharmacokinetic properties imparted by the silane, DB-67 was deployed in xenograft models, where potent tumor growth inhibition was observed (61% and 73% after five days at 3 and 10 mpk daily dosing), and full regression was achieved at 21 days of an elevated dose (30 mpk/day).

Further development of this concept led to the discovery of Karenitecin (**6.5**), a synthetic analog of camptothecin bearing an ethyl silane at the C7 position, initially developed by Bionumerik Therapeutics. This compound was engineered to achieve higher lactone concentration in plasma and designed with a hydrogen at the C-10 position rather than a hydroxyl group, thereby circumventing unnecessary hepatic glucuronidation and bypassing mechanisms of unfavorable metabolism.

**Figure 6:**
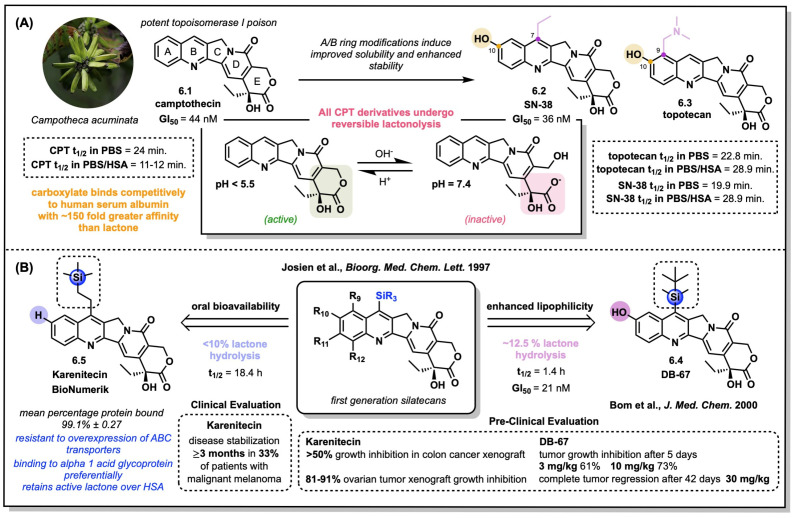
Karenitecin and DB-67 are potent Topoisomerase I poisons with improved lipophilicity and bioavailability. (**A**) A/B ring modifications on camptothecin show increased stability of the E-ring lactone pharmacophore yielding FDA-approved drugs SN-38 and topotecan. (**B**) The installation of organosilicon containing side chains at C7 in DB-67 and karenitecin has led to increased lipophilicity, blood solubility, and show prominent results in pre-clinical and clinical studies [54,100].

Kareniticin (**6.5**) exists in plasma as 90–95% as the lactone form, while DB-67 exhibits marginally higher hydrolysis, with 87.5 ± 8.5% of the drug remained in the lactone form, which may play a role in the differences in the clearance half-life of these compounds [101,102] (Figure 6).

Preclinical studies demonstrated substantial antitumor activity across multiple xenograft models, including ovarian and colon cancers, with efficacy comparable to or exceeding topotecan. Karenitecin retains activity in multidrug-resistant cell lines characterized by overexpression of P-glycoprotein and BCRP efflux transporters [103]. In Phase II clinical studies, it was demonstrated that the dosing of Karenitecin-induced disease stabilization in 33% of patients with metastatic melanoma lasting for ≥3 months [104]. Combined with the drug’s enhanced stability and increased lipophilicity, it was evaluated for the treatment of gliomas and other CNS malignancies primarily due to BBB permeability. Although the drug exhibits relatively poor CNS penetration (5%), improved bioavailability correlated to higher amounts of the predominant lactone in plasma 90% and a half life of 18.4 h marking a significant improvement over conventional camptothecins [102].

Mechanistically, the improved pharmacokinetic profile of karenitecin (**6.4**) has been attributed not only to increased lipophilicity, but also altered plasma protein binding. In contrast to earlier camptothecin derivatives, which preferentially associate with human serum albumin (HSA), karenitecin (**6.4**) exhibits competing binding to α1-acid glycoprotein (AAG), which is lower in natural abundance than HSA in human serum samples. Smith and coworkers elucidated that Karenitecin was 99.1% plasma-bound, directly proportional to the concentration of AAG present in the sample. Remarkably, the compound maintains its strong affinity for the AAG protein despite abundant plasma concentrations of HSA (35–50 mg/mL) compared to AAG (0.5–1.0 mg/mL) [101]. This redistribution of protein binding may contribute to the stabilization of the active lactone form and prolonged systemic exposure, although the precise interplay between protein binding and lactone hydrolysis equilibria remains an active area of investigation. Collectively, these studies highlight the utility of silicon incorporation as a strategy for augmenting the pharmacological properties of camptothecin derivatives and analogs. By influencing lipophilicity, metabolic stability, and protein binding equilibria, the addition of arylsilyl substituents provide a means of addressing fundamental limitations associated with the camptothecin scaffold, ultimately enabling improved therapeutic performance as an anticancer agent.

### 2.5. Silanes in Retinoic Acid Receptor (RAR) Agonists

**Figure 7:**
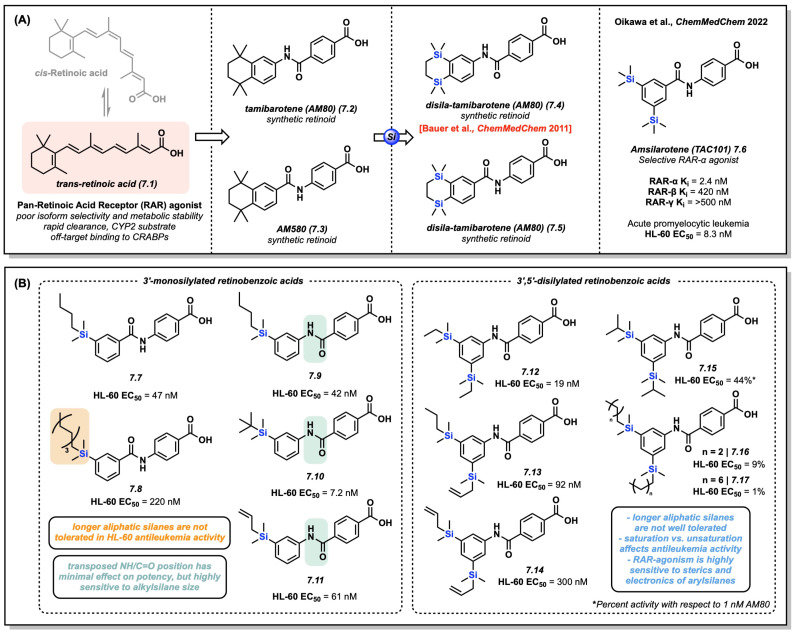
Retinoic acid receptor (RAR) agonists as differentiation-inducing antileukemia agents. (**A**) *Trans*-retinoic acid, the endogenous ligand to RAR receptors, has pan-RAR activation profiles but limited clinical applicability. This has led to the design of synthetic retinoids Tamibarotene (AM80) and its analog AM580, both of which have more desirable physiochemical properties and pharmacokinetic profiles, but which do not achieve isoform selective RAR activation; and TAC101, a biaryl retinobenzoic acid with impressive RAR-α isoform selective agonistic activity [109,110]. (**B**) The diversity of arylsilanes installed on the retinobenzoic acid scaffolds has led to several notable trends in arylsilane reactivity. The installation of a monosilyl group at the 3′ position retains activity, though in both that system and in its benzamide transposed isomer, a consistent theme of lowered potency as a function of aliphatic chain length emerges.

The highly sensitive nature of lymphocyte proliferation to retinoic acid receptors (RARs), which function to regulate lymphocyte differentiation, led to the initial discovery from Mark Breitman and coworkers [105,106] that retinoic acid **7.1**, the native agonist to all RAR isoforms, can induce terminal differentiation of myeloid leukemia cells into granulocytes, apprehending their proliferation and resulting in a diminishing population of circulating myeloid cells. This process effectively relieves the differentiation block characteristic of acute promyelocytic leukemia (APL), resulting in the maturation of leukemic promyelocytes rather than their direct cytotoxic elimination. Clinically, this strategy has proven highly effective in APL, where malignant cells are particularly sensitive to RAR-α mediated transcriptional programs that restore normal differentiation. However, the poor biophysical and metabolic profile of *trans*-retinoic acid, including rapid clearance, CYP-mediated olefin oxidation, off-target binding to cellular retinoic acid binding proteins (CRABPs), and known *trans-/cis*-isomerism that may lead to off-target binding has necessitated the development of synthetic small molecules with more desirable metabolic stability and isoform selectivity. This has subsequently led to the discovery of several small molecules with potent RAR-agonist properties, including Tamibarotene (AM80, **7.2**) and its isomer AM580 (**7.3**), which retained potent RAR agonist activity but exhibited far better metabolic and bioavailability profiles in comparison to retinoic acid [107,108] (Figure 7).

Recent work by Bauer et al. deployed a single atom substitution strategy in the preparation of dilsila-tamibarotene and its isomer (**7.4** and **7.5**, respectively) which exhibited modest improvements in RAR potency but still suffered from poor isoform selectivity [109]. TAC101 (**7.6**) and its analogs have demonstrated remarkable RAR-α selectivity (K_i_ = 2.4 nM) over RAR-β and RAR-γ (K_i_ = 420 nM and >500 nM, respectively), and in vitro antileukemia activity in HL-60 cells (EC_50_ = 8.3 nM). Further elaboration of these silylretinobenzoic acids in the 1990’s and in the more recent literature have led to more detailed SAR on the role of alkyl substituent and positioning of the arylsilane group [110]. Analogs with just one of the two silane groups ***7.7*** and **7.8** revealed that while small aliphatic silanes at C3′ retained some antileukemia activity in HL-60 cells, lengthier aliphatic groups such as the *n*-octyl alkyl chain on **7.8** led to a precipitous loss of potency. This SAR published by Oikawa et al. [110] also demonstrated that repositioning of the benzamide connection between the two rings did not significantly affect potency (compounds **7.9–7.11**) but that even minor changes to the alkylsilane motif present can have significant perturbations on HL-60 antileukemia activity. For example, *tert*-butyldimethylsilane **7.10** exhibits markedly greater potency (EC_50_ = 7.2 nM) comparable to TAC-101, but its structural isomer **7.9** with an n-butyldimethylsilane is significantly less active (EC_50_ = 42 nM). When compared against a broader library of 3′5′-disilylated aryl functionalities, the observed trend of diminishing antileukemia activity with longer aliphatic chain lengths is consistent with these earlier trends. Taken together, these studies highlight the sensitivity of RAR ligand activity to subtle steric and electronic perturbations introduced by silicon substitution. While silicon incorporation can be used to fine tune potency and receptor selectivity, these effects are highly dependent on substituent size and positioning, underscoring the importance of precise structural control in the design of silicon-containing retinoids.

### 2.6. Silylporphyrins as Photosensitizers

Porphyrins, heterocyclic macrocycles critical towards various bioenergetic processes, have been additionally exploited in the clinic for their photoelectric properties imbued by an extensive conjugated π-system [111,112]. Beginning with the discovery of hematoporphyrin by Scherer in 1841, porphyrin-based photosensitizers have garnered widespread investigation in the development of photodynamic therapy (PDT), a cytotoxic strategy involving radiation-induced in situ generation of singlet oxygen (^1^O_2_) [111,113,114]. Over the years, structural modifications to the porphyrin scaffold have led to the development of several derivatives, including tetraphenylporphyrin (TPP) and sulfonate compound TPPS_4_ [115,116], which demonstrated improved stability, singlet oxygen quantum yields, and antiproliferative activity while retaining fundamental photophysical properties of prior porphyrin-based photosensitizers.

**Figure 8:**
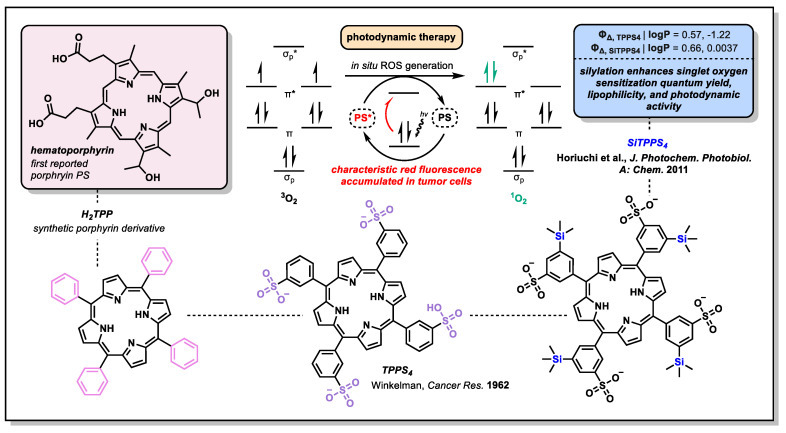
Development of silylated porphyrin photosensitizers with greater phototoxic activity. Porphyrins are privileged scaffolds in the photodynamic therapy (PDT) space due to their electron rich aryl systems, which enhance photosensitizer triplet state quenching and singlet oxygen generation. Localization of such compounds in tumor cells has been characterized by a red fluorescence attributed to photosensitizer relaxation concomitant with singlet oxygen generation. Over the years, various hydrocarbon- and heteroatom-based derivatives of this structure, including TPP and TPPS4, have been synthesized and found to improve sensitization efficacies [119]. More recently, Horiuchi and coworkers revealed silylation of TPPS4 as another strategy to increase singlet oxygen quantum yield and antiproliferative activity [117,118]. The asterisk (*) in the molecular orbital diagram indicates an antibonding orbital.

Previously, Horiuchi and coworkers prepared trimethylsilyl-substituted porphyrin SiTPP and demonstrated that it enhanced photosensitization significantly from parent compound TPP [117,118]. The corresponding sulfonate version (SiTPPS_4_) was subsequently designed and evaluated for its electronic and antiproliferative properties [116] (Figure 8). Expectedly, SiTPPS4 exhibited a sizable increase in singlet oxygen quantum yield (ɸΔ = 0.66) and sensitization efficiency (f_Δ_^T^ = 0.76) from TPPS4 (ɸΔ = 0.57, f_Δ_^T^ = 0.66) while maintaining comparable rate constants of singlet-triplet interconversion and internal conversion. The augmented photoelectric effects of silylation exclusively on singlet oxygen generation render SiTPPS4 a promising candidate for PDT from a molecular standpoint.

In vitro evaluation of SiTPPS_4_ and TPPS_4_ in U251 human glioma cells revealed that both compounds displayed minimal cytotoxicity at lower concentrations in the absence of irradiation, although the silylated derivative led to modest killing beyond 50 μM. Notably, upon visible light irradiation, SiTPPS_4_ exhibited significant phototoxic activity beyond what was proportional to an enhanced ɸΔ. In investigating this phenomenon, it was found that despite the comparable fluorescence quantum yields of both sulfonate compounds, SiTPPS_4_ emitted a greater red fluorescence intensity in live-cell imaging. The authors attributed this to the improved lipophilic character of the silylated porphyrin (logP = 0.0037) over TPPS_4_ (logP = −1.22), which would enable more efficient cellular uptake of the photosensitizer. These findings exemplify the unconventional application of silanes and their electron-donating nature as a means to improve both bioavailability and electronic properties, opening an additional door for bioorthogonal investigation in PDT.

### 2.7. A Silyl Spacer: Combretastatin A4

**Figure 9:**
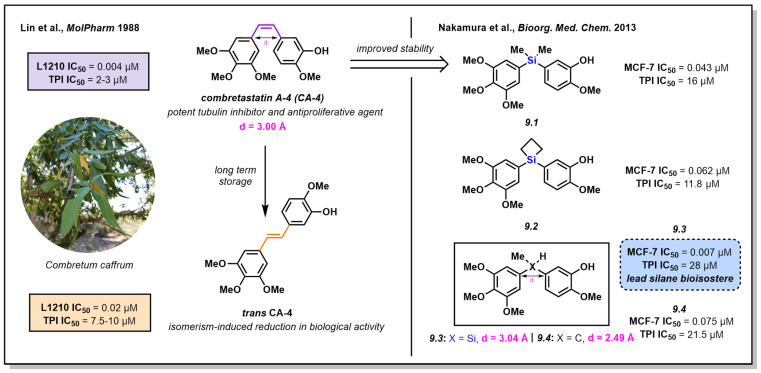
Silane-based linkers to mitigate loss of potency caused by *cis–trans* isomerization in combretastatin A-4. Combretastatin A-4 (CA-4), a potent natural product inhibitor of tubulin at the colchicine site, suffers from a loss in activity as a result of isomerization to a more stable *trans*-isomer [120]. Nakamura and coworkers sought to ameliorate this through the installation of bioisosteric silane linkers in place of the alkene moiety, wherein **9.3** was observed to perform the most proximally to CA-4 as an antiproliferative agent [130]. (Picture of *C. caffrum* by Christiaan Viljoen is licensed under Creative Commons CC-BY, Wikimedia Commons).

Silicon-containing groups, owing to their atomic dimensions, have been further explored in medicinal chemistry as potential spacers with enhanced physicochemical properties. The C-Si bond, which is longer than carbon-carbon or carbon-heteroatom bonds commonly deployed in small molecule drug scaffolds (Table 1), offers a stable alternative towards conventional linker systems whose torsional strains may impede upon a molecule’s durability and biological activity. Bioisosteric strategies involving the installation of this bond have been employed in the combretastatin space, a family of natural products first isolated from *Combretum caffum* in the 1980s [120]. Among them, combretastatin A-4 (CA-4) has been of particular interest as a nanomolar antiproliferative agent and inhibitor of the colchicine tubulin binding site [121,122,123]. The efficacy of this compound is known to be limited by its isomerism over time to a stabler but significantly less potent *trans*-form (Figure 9), which has led to pharmacophore editing of the olefin as an emerging approach in mitigating this liability [120,124,125,126,127,128,129].

For example, Nakamura and coworkers investigated the usage of silanes as durable linkers between the two aryl fragments on CA-4 [130] (Figure 9). The insertion of a singular dimethylsilyl group, corresponding to compound **9.1**, was found to span a comparable distance (d = 3.03 Å) to the ethylene moiety (d = 3.00 Å) in silico, and docking results suggested key tubulin binding interactions of this silylated analog that mimicked those of CA-4. Accordingly, a library of silane-functionalized CA-4 analogs was synthesized and evaluated in MCF-7 cell proliferation, tubulin polymerization inhibition (TPI), colchicine competitive binding, and physicochemical stability assays. Bulkier silanes, such as those bonded to ethyl, isopropyl, or ethoxy groups, correlated to markedly diminished inhibition of tubulin and antiproliferative activity. The relation between sterics and potency with regards to MCF-7 cell viability was similarly observed in the lead compounds **9.1** (IC_50_ = 0.043 µM), **9.2** (IC_50_ = 0.062 µM) bearing a silacyclobutane, and monomethylated **9.3** (IC_50_ = 0.007 µM), which displayed particularly proximal potency to CA-4 (IC_50_ = 0.004 µM). This trend in antiproliferative activity, however, does not align with that of the observed tubulin inhibition, wherein **9.2** (IC_50_ = 11.8 µM) demonstrated the most comparable inhibitory effects to CA-4 (IC_50_ = 4.5 µM), and compounds **9.1** (IC_50_ = 16.0 µM) and **9.3** (IC_50_ = 28.0 µM), despite their greater activity in vitro consistent with reduced steric hindrance, were notably less effective in this cell-free assay. Interestingly, the silacyclobutane seems to fall into the “goldilocks” zone of tubulin inhibition, and either an increase or decrease from its sterics were less tolerable. This was reflected in the inhibition of colchicine binding, where **9.2** exhibited remarkable inhibition (88.1%) at 3 µM, albeit compound **9.3**, which performed the best in cell proliferation assays but suboptimally in TPI measurements, demonstrated superior competitive inhibition of 90.7% at the same concentration. These findings suggest that biarylsilanes perturb structure-function relationships beyond simply acting as a silane-based spacer bioisostere. A precipitous loss in potency of the corresponding monomethylated carbon compound **9.4** across MCF-7 cell proliferation and the two pharmacodynamic assays further confirmed the unique properties of silicon that enable its potential as a substituted linker. Additionally, while the parent combretastatin CA-4 isomerized in PBS over time, the silane analogs **9.1** and **9.3** retained their geometric integrity and biological potency, corroborating this replacement of a *cis*-stilbene with a chiral silane. Altogether, this natural product study introduces the prospect of employing silanes not only for favorable binding site interactions, but also as bioisosteres of cis-stilbenes that ameliorate *cis–trans* isomerization-induced loss of potency.

### 2.8. Silanes in STS Inhibitors

The development of silicon-incorporated steroid sulfatase (STS) inhibitors represents a strategic shift in the design of therapeutic agents for postmenopausal hormone-dependent breast cancer [131,132,133,134,135]. The parent compounds for this study are non-steroidal diphenylmethane (DPM) derivatives, which were selected to circumvent the inherent optimization challenges and potential off-target effects associated with traditional steroidal scaffolds. The primary compound acts as a potent inhibitor of the STS enzyme, thereby preventing the conversion of inactive steroid sulfates into tumor-promoting estrogens, and is subsequently hydrolyzed by the enzyme to release bisphenol metabolite. Critically, these metabolites are engineered to possess estrogen receptor α-antagonistic activity, ensuring that any metabolic byproduct further suppresses tumor growth rather than inadvertently stimulating it through agonistic effects. To optimize this dual-action profile, Kajita and coworkers focused on the central scaffold of the DPM skeleton, identifying a silicon-for-carbon substitution as a means to fine tune both enzymatic inhibition and receptor affinity [131].

**Figure 10:**
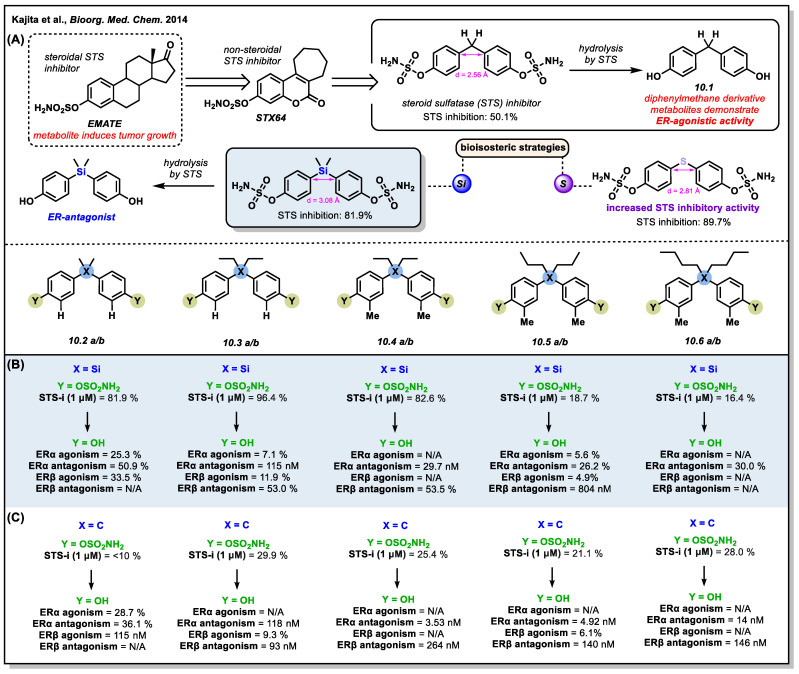
Silicon-containing diphenylmethane derivatives were synthesized as non-steroidal steroid sulfatase (STS) inhibitors [131]. (**A**) Substitution of the central carbon on the diphenylmethane scaffold with silicon increases the distance between the two benzene rings from 2.56 Å to 3.08 Å, possibly allowing for improved binding affinity within the STS enzyme active site. (**B**) Silicon-containing derivatives demonstrated superior STS inhibitory potency relative to their carbon analogs. The silane metabolites were shown to act as potent ERα antagonists with increased selectivity for ERα over ERβ. (**C**) STS inhibitory potency in carbon-containing STS analogs, with lower overall ER agonist activity compared to their silylated counterparts.

Computational modeling and X-ray crystallographic analysis revealed that substituting the central quaternary carbon of compound 10.0 with silicon increases the distance between the two benzene rings from 2.56 Å to 3.08 Å (Figure 10). This structural expansion, combined with the higher electropositivity of silicon, was hypothesized to facilitate a more favorable orientation within the STS enzyme’s active site and improve the binding affinity of the resulting silane-bridge derivatives.

Experimental data confirmed that the introduction of silicon yielded several significant pharmacological advantages, though it also highlighted specific structural constraints. Silicon-containing derivatives, such as compound **10.4a**, demonstrated superior STS inhibitory potency (IC_50_ = 0.17 μM) relative to their carbon analogs; however, they did not perform better than their sulfur counterparts (Figure 10). It was identified that replacing the methyl groups on the center silicon atom with ethyl increased STS inhibitory activity from 81.9% to 96.4%. Subsequent studies on ethyl, propyl, and butyl analogs **10.4a**, **10.5a**, and **10.6a** demonstrate that bulkier substitutions reduce STS inhibition.

This trend is consistent with ERα antagonistic activity wherein larger alkylsilanes exhibit a precipitous loss in activity. Furthermore, the silane metabolites exhibited a highly desirable biological profile, acting as potent ERα antagonists with increased selectivity for ERα over ERβ, and entirely lacking the estrogenic agonism seen in earlier lead compounds. These results underscore the profound impact of single-atom substitution within the DPM skeleton, where the replacement of a central carbon with silicon serves as the molecular trigger for enhancing both inhibitory potency and antagonistic character.

### 2.9. Silylated Vitamin D Receptor (VDR)

The vitamin D receptor (VDR) is a ligand-dependent transcription factor that plays a critical role in various physiological processes, including immune response, cell differentiation, and calcium homeostasis [136]. Given the role of VDR dysfunction to diseases such as psoriasis, osteoporosis, and several cancers, hundreds of secosteroid-based ligands have been developed, with some currently approved for clinical use. The development of non-secosteroidal ligands is expanding, considering the expectation that these novel core structures are expected to offer improved metabolic and chemical stability while reducing the risk of side effects [137,138]. Research into this space has identified several potent chemotypes, such as diphenylmethane and diphenylsilane derivatives, leading investigators to explore the systematic impact of the hydrophobic core on ligand activity. The investigation into silicon incorporation was specifically motivated by its advantages over carbon.

**Figure 11:**
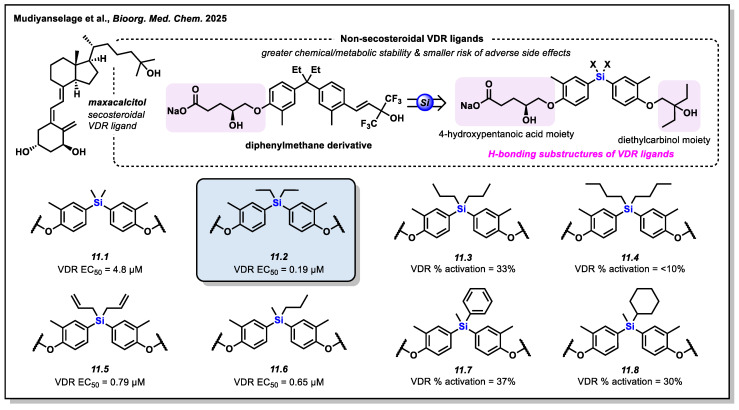
Silyl substitutions on diphenylmethane derivatives as non-secosteroidal VDR ligands have been hypothesized to impact compound activity by enhancing hydrophobic interactions and allowing for sterically crowded quaternary centers. The activity of these diphenylsilane motifs are highly sensitive to the length and bulkiness of the substituents on the silicon atom [136].

The systematic investigation of diphenylsilane derivatives as VDR ligands reveals that the incorporation of silicon significantly impacts compound activity by enhancing hydrophobic interactions and allowing for sterically crowded quaternary centers (Figure 11). Silicon’s increased hydrophobicity compared to carbon is advantageous for promoting these hydrophobic interactions within the VDR’s ligand-binding domain. The structure–activity relationship (SAR) studies described by Mudiyanselage and coworkers identified compound **11.2**, a diethyl-di-*m*-tolylsilane derivative, as the most potent agonist with an EC_50_ of 0.19 μM. This demonstrated that the small molecule’s activity is highly sensitive to the length and bulkiness of the substituents on the silicon atom. For example, extending the alkyl groups to di-*n*-propyl (**11.3**), or di-*n*-butyl (**11.4**), or truncation of the dialkyl groups to the dimethylsilane (**11.1**) led to a marked decrease or total loss of agonistic activity. Interestingly, the diallylsilyl derivative **11.5** maintained significant activity (EC_50_ = 0.79 μM) despite having a similar chain length to the less potent *n*-propyl version, indicating that small structural or electronic differences can greatly influence ligand-receptor interactions [136].

Crystallographic analysis of the fourteen diphenylsilane compounds confirmed that potent agonists consistently form hydrogen bonds with residues His301 and His 393. This revealed a stereochemical preference for the Si-*(R)*-configuration in asymmetric compounds, such as Compounds **11.6** and **11.7** which was identified as the favorable arrangement for VDR transactivation activity. Given the close contacts between the dialkyldiarylsilane linker and Trp282, the diethylsilane appears to have the optimal tradeoff of steric demand and size with productive hydrophobic association with Trp282. Conversely, the lack of activity in certain compounds, such as the di-*n*-propyl derivative (**11.3**), was attributed to an unfavorable ligand conformation that imposes a significant conformational energy barrier during binding. These substituents were predicted by the authors to cause unfavorable changes in allosteric effects, receptor cofactor dynamics or cause crystallographic limitations regarding coactivator recruitment and transcriptional activation. Ultimately, Mudiyanselage and coworkers conclude that while hydrophilic and hydrophobic interactions are critical, the specific ligand conformation within the receptor is a decisive factor in determining transcriptional activity [136].

### 2.10. Miscellaneous Applications of Organosilane Functionalized Bioactive Compounds

Beyond the aforementioned examples, this strategy has been applied to other bioactive scaffolds outside of the cancer discovery chemistry space, including sila-Ibuprofen [139], sila-canniboids [140], indomethacins [141], brain-penetrant oxazolidinones [142], rimonabant [143], venlafaxine [144,145], and heme-binding indazoles [146], among others. While these studies target important clinical indications apart from cancers, they fall outside of the scope of the present review.

## 3. Silyl Ethers

**Figure 12:**
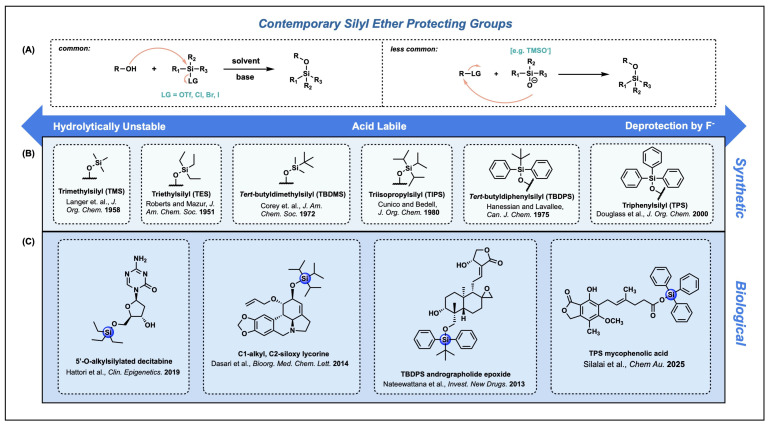
Contemporary silyl ether protecting groups reimagined as biological protecting groups (**A**) Silyl ether protecting groups can be installed on alcohols via two generalized routes, the first involving a nucleophilic attack by a hydroxyl onto a trialkylsilyl leaving group (such as a halide or a triflate), and another, less common method involving a trialkylsilenoate as a nucleophile. (**B**) Silyl ethers range in their reactivities, from hydrolytically unstable groups such as TMS to groups that can be deprotected by fluorine, such as TPS [32,147,148,149,150,151,155]. (**C**) The characteristics of these silyl-protecting groups mirror their activity on biologically active scaffolds [156,157,158,159].

For nearly seventy years, silyl ethers have been exploited in synthetic organic chemistry as both reactive intermediates and as protected alcohols. From initial observations that trimethylchlorosilane (TMSCl) provided expedient access to silyl ethers from alcohols reported by Irving Wender in 1958 [147], to Corey’s seminal innovation of bulkier and more hydrolytically stable *tert*-butyldimethylsilyl ethers (TBDMS) groups as protecting groups for alcohols [32], the contemporary array of alcohol silylating reagents that are now commercially available have been enabling in the installation of silyl ethers with carefully tuned reactivities (Figure 12B). The intentional selection of silyl ethers as protecting groups has been particularly enabling in the total synthesis of complex natural products involving multiple hydroxyl substituents with competing reactivities [33,148,149,150,151].

This structural diversity, arising from the breadth of commercially available silylating reagents, provides a modular platform for systematically tuning steric and electronic properties, thereby enabling opportunities for structure-activity relationship exploration in silyl ether-containing scaffolds. Further, these same reactivity trends translate into a range of biological functions of silyl ethers that span from hydrolytically labile prodrugs that can desilylate at will to hydrolytically robust silyl ether groups whose metabolic durability is a function of their alkyl substituents (Figure 12C) [152,153,154]. This capacity to modulate silyl ether stability offers a versatile handle for tuning drug exposure and activation in anticancer contexts. To this end, the application of silylated hydroxyls on natural product scaffolds is an emerging area of not only synthetic adaptability but also a means to interrogate biological function.

### 3.1. Silyl Ether Analogs of Withaferin A

A diverse array of C-27 and C-4 silyl ether analogs of withaferin A, a bioactive steroidal lactone derived from the *Withania somnifera* plant with known anticancer activity, was evaluated by Perestelo and coworkers as a potential strategy to access more desirable drug-like properties and physicochemical profiles [160,161].

Previously, WA (**13.1**) has been studied for its ability to modulate diverse signaling pathways, including NF-κB, STAT3, and the induction of oxidative stress, primarily acting as a pro-oxidant to trigger apoptosis and DNA damage in various cancer cells [162,163,164,165,166]. The synthesis of silyl ether derivatives represents a generalized trend toward improving in vitro cytotoxicity. However, the primary mechanism through which these analogs function remains complex, where some compounds appear to induce classical apoptotic cell death, and others exhibit distinct cell cycle arrest profiles, suggesting that siloxy-modification induces a mechanistic divergence from the parental scaffold. While WA typically triggers G2/M phase arrest, the most potent silyl analog, containing a *tert*-butyldimethylsilyl at the C-27 position, induces significant apoptosis without a corresponding cell cycle arrest, indicating a shift in the primary molecular target or the activation of alternative programmed cell death pathways [160].

**Figure 13:**
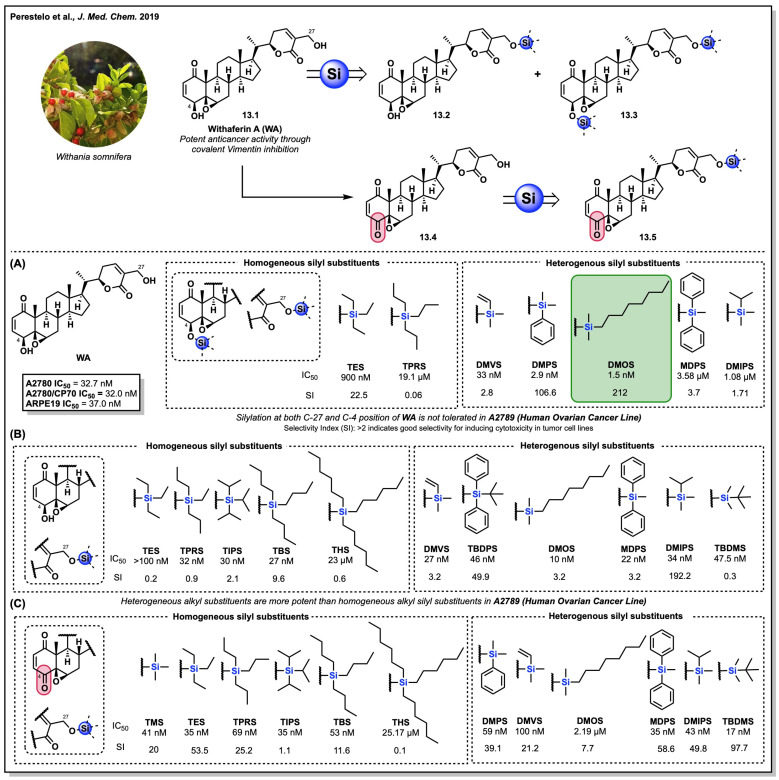
Incorporation of silyl ethers on withaferin A C4 and C27 positions alter potency in Human ovarian cancer cells and cytotoxic selectivity [160]. (**A**) Silylation at both C27 and C4 position of WA 13.1 is not well tolerated in A2789 human ovarian cancer cells. (**B**) Heterogeneous alkyl substituents are more potent than homogeneous alkyl silyl substituents. (**C**) Modification at C27 coupled with a carbonyl group at C4 significantly enhanced the drug-like profile and cytotoxic efficacy of these compounds (photograph of *W. somnifera* by Biswarup Ganguly, Wikimedia Commons, published under Creative Commons CC-BY).

A critical observation in the structure–activity relationship was the superior cytotoxic performance of heterogeneous alkyl substituents (TMS, TES, TPRS, TIPS, TBS, THS) over homogeneous alkyl silyl substituents (DMVS, DMPS, DMOS, MDPS, DMIPS, TBDPS, TBDMS) in A2780 human ovarian cancer cell lines, where these unsymmetrical silyl ethers generally exhibited higher potency, which the authors attribute to a more favorable balance of lipophilic and steric accessibility (Figure 13).

Distinct potency trends were observed, favoring C-4 modification on WA over the C-4 and C-27 double-silylation. Modifications at the C-27 position, particularly combined with the presence of a carbonyl group, significantly enhanced the drug-like profile and cytotoxic efficacy of these compounds in human ovarian cancer cells. This trend was further validated in the cisplatin-resistant A2780/CP70 engineered cell line, where several C-27 silylated analogs, notably, TPRS (IC_50_ = 3.6 nM), exhibited single-digit nanomolar potency, effectively bypassing the resistance mechanisms that render traditional platinum-based chemotherapy drugs ineffective. Specifically, while the parent WA showed an IC_50_ of 32.0 nM, several silylated analogs maintained a near-equipotent or greater potency profile in the resistant lines, such as the dimethyl octyl silyl ether, that demonstrated an IC_50_ of 1.5 nM in the A2789 cell line, suggesting that this strategy improves potency even against cisplatin resistant cell lines.

Crucially, these analogs demonstrated remarkable selectivity. When evaluated against noncancerous ARPE19 (human retinal epithelial) cells, potent silyl ether analogs, such as the DMOS, maintained a high selectivity index (SI) of 212 compared to natural product WA, with an SI of 1.1. This suggests that the silicon-based lipophilicity tuning can allow for preferential sensitivity for cancer cells while sparing healthy tissue, which is a vital requirement for transitioning from a natural lead to a viable clinical candidate.

### 3.2. Silyl Ether Analogs of Andrographolide: New Structures and New Mechanisms

A similar strategy has been deployed in analogs of andrographolide, a labdane diterpenoid isolated from the herb *Andrographis paniculata* which has been used in traditional medicines for its anti-inflammatory properties [167,168,169]. This molecule, which putatively functions through inhibition of the transcription factor NF-κB [170,171,172,173], has been explored for clinical applications in inflammation [174,175], cancer, and neurodegenerative disease [176,177]. While this compound has demonstrated a very broad range of potential therapeutic applications, its poor oral availability and proclivity towards metabolism and clearance (t_½_ = 4.6 h) limit its clinical and preclinical success. Tran and coworkers reported that glucuronidation and sulfation at the C19 and C3 hydroxyls are major metabolic pathways of the in vivo clearance of andrographolide [167,178].

**Figure 14:**
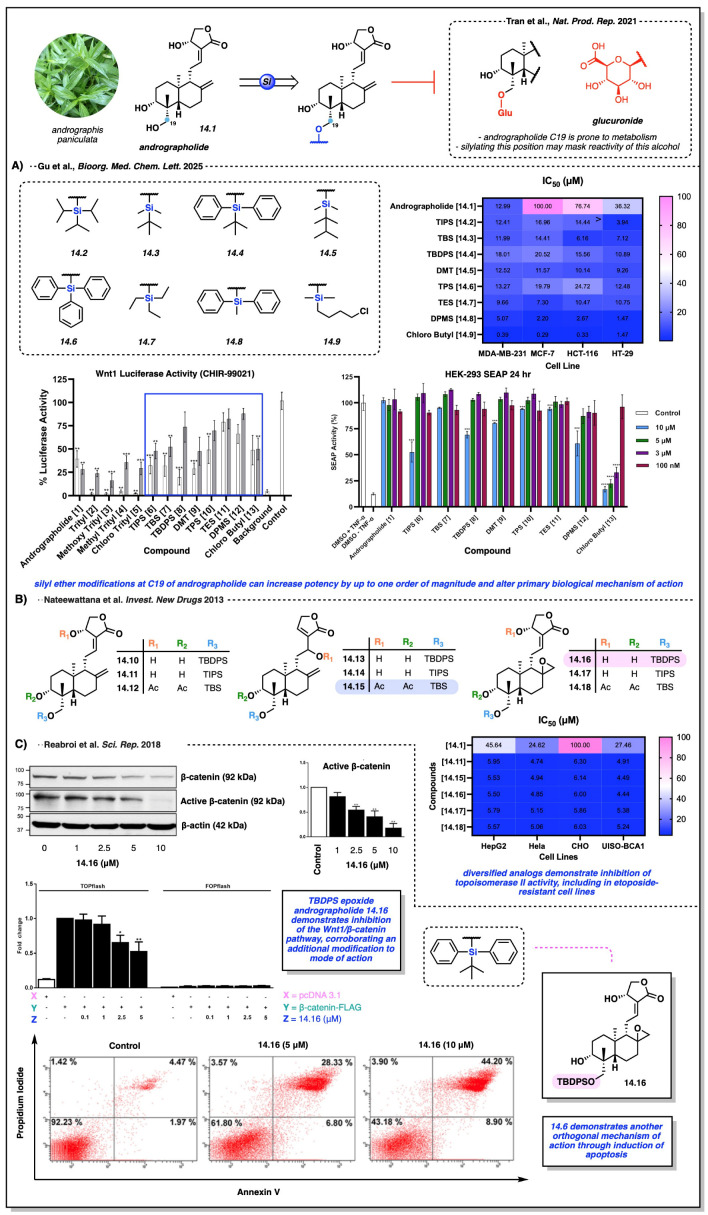
Installation of silyl ethers on a diterpenoid scaffold Andrographolide increases potency and modulates mechanism of action [167]. (**A**) Biological evaluations of a silyl ether library indicate an increase of potency of andrographolide analogs by one order of magnitude and dose-dependent inhibitive activity in NF-κB and Wnt1/β-catenin reporter cell assays. Statistical significance was determined using a Welch’s *t*-test (n = 3) (** *p* < 0.01, *** *p* < 0.001, **** *p* < 0.0001). (**B**) Andrographolide with modifications on C19, C3, C17, C12, and C14 demonstrate a substantial increase in potency in Hep02, HeLa, CHO, and UISO-BCA1 cell lines through topoisomerase II inhibition [158]. (**C**) The effects of analog **14.16** were further quantified with flow cytometry, suggesting that analog **14.16** induced apoptosis as opposed to generalizable cell death; TOPflash and FOPflash essays, suggesting Wnt1/β-catenin inhibition (* *p* < 0.05, ** *p* < 0.01); and western blot, indicating a substantial decrease of active β-catenin (parts of figure adapted from references Gu, et al. Bioorg. Med. Chem. Lett. 2025 ([179]) and Reabroi, et al. Sci. Rep. 2018 ([180]) with permission. © Springer Nature/Elsevier).

Our laboratory, leveraging the diverse accessibility of silicon-containing alcohol protecting group reagents that are conventionally used in organic synthesis, re-envisioned their application as biological protecting groups to mask the reactivity of the primary C19 hydroxyl group [179]. A library of twelve tritylated and silylated compounds was synthesized to further investigate the role of hydrophobic substitutions at C19. (Figure 14A) Silyl ether analogs **14.2**–**14.9** exhibited about an order of magnitude greater anticancer potency relative to andrographolide. Interestingly, these compounds exhibited a higher potency in colorectal cancer cell lines (HCT-116, HT-29) compared to breast cancers (MDA-MB-231, MCF-7). Further, a firefly luciferase reporter cell assay was used to interrogate the role of andrographolide silyl ether analogs in Wnt1 modulation, where we observed potent dose-dependent inhibition of Wnt1/β-catenin-dependent fLuc expression compared to the minimal activity displayed by the parent compound. Moreover, to quantify activity of these analogs in NF-κB inhibition, we utilized a TNF-α activated secreted alkaline phosphatase (SEAP) reporter cell assay, and found that several compounds elicited significant inhibition of SEAP expression, albeit only at the highest concentrations (10 μM). Collectively, these observations indicate that the installation of silyl ethers at C19 of andrographolide may re-orient the biological targets of andrographolide and its analogs.

Consistent with the increased potency of C19 silyl analogs we reported, Nateewattana et al. identified that C-19 silyl ether analogs of andrographolide that additionally contain functionalization at C3, C17, and C14 (Compounds **14.10**–**14.18**) exhibit far more potent antiproliferative activity against HepG2, HeLa, and BCA-1 cancer cell lines compared to the parent compound andrographolide [158] (Figure 14B). The authors further utilize a biochemical DNA-cleavage assay to identify how these compounds may impact fundamental mechanisms of nucleic acid replication, and propose that these analogs function through potent inhibition of topoisomerase II. In direct comparison with a topoisomerase II inhibitor etoposide (IC_50_ = 46.77 μM), compounds **14.11** (IC_50_ = 6.30 μM), **14.15** (IC_50_ = 6.14 μM), **14.16** (IC_50_ = 6.00 μM), **14.17** (IC_50_ = 5.86 μM), and **14.18** (IC_50_ = 6.03 μM) retained potency, demonstrating surprising toxicity in etoposide-resistant CHO cells. This observation indicates that these analogs may function through mechanisms independent of topoisomerase II inhibition. This is consistent with the broader SAR of silyl ethers of andrographolide reported earlier by Sirion et al. where silyl ethers, among the A-ring acetonide and trityl ethers, exhibited marked improvement in anticancer potency in comparison to the natural product itself [181]. Thus, Reabroi and coworkers [180,182], recognizing the TCF4 crosslink between topoisomerase II and the oncogenic Wnt1/β-catenin signaling axis, demonstrated in colorectal cancer HT-29 cells that analog 13.16 with a tert-butyl diphenyl silyl ether (TBDPS) on C19 and a B-ring epoxide elicits its anticancer activity through potent inhibition of the Wnt1/β-catenin pathway, which was validated through positive signal on a TOPFlash luciferase assay and through reduction of phosphorylated β-catenin by Western Blot (Figure 14C). Further, the authors posit that the retention of activity with lithium co-administration suggests a GSK-3β independent mechanism of action.

Notwithstanding the mechanistic ambiguity through which C19-functionalized andrographolide analogs have been purported to function through, our laboratory and others have collectively observed both increases in anticancer potency and significant shifts in the functional outcomes of more detailed mechanistic interrogation of C19-silyl andrographolide structure–activity relationships.

### 3.3. Lycorine

In another example of the convenient and modular structure–activity relationships offered by silyl ethers in modulating the lipophilicity of drug-like molecules, Dasari et al. utilize this strategy to functionalize lycorine, a molecule isolated from the species *Amaryllidacaea* [157,183,184,185,186]. Lycorine putatively functions by binding to eukaryotic translation elongation factor 1A (eEF1A) and disrupting cytoskeleton organization [183]. Remarkably, this compound maintains anticancer activity against cells that are resistant to pro-apoptotic drugs, and displays significant activity in cancer cells over non-cancer cells, demonstrating its appeal as a therapeutic drug scaffold.

**Figure 15:**
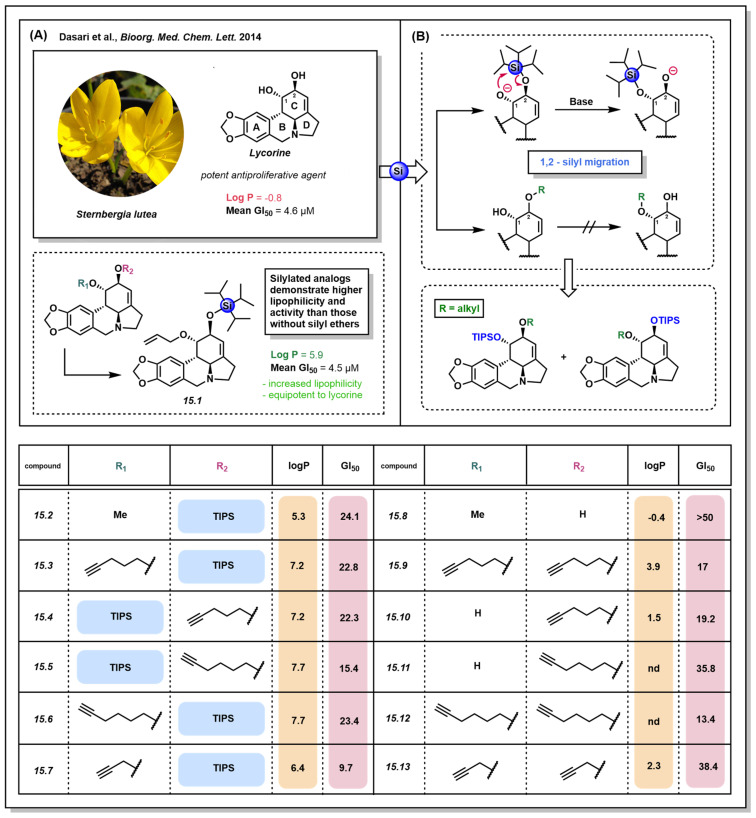
Inclusion of a triisopropylsilyl protecting group on either the C1 or C2 positions of lycorine greatly increases lipophilicity and the regularity of anti-cancer activity [157]. (**A**) Compound **15.1**, with a TIPS group at the C2 position and an alkene at the C1 position displays multiple orders of magnitude of increase in the lipophilicity of lycorine while maintaining similar potency. (**B**) Under basic conditions, the TIPS group on lycorine can migrate between C1 and C2 positions, while the same trend cannot be observed for alkyl substituents, suggesting a mechanistic difference that the longer Si-O bond plays in comparison to the analogous C-O bond in a typical ether (photograph of *S. lutea* by M. Martin Vicente, Wikimedia Commons, published under Creative Commons CC-BY).

To explore the consequent mechanism of action of silylation at the C-ring anti-diol of lycorine, Dasari et. al. prepared and evaluated a panel of C1- and C2-silyl ethers among several other analogs in cancer lines that were either apoptosis-sensitive or showing resistance to apoptosis [157]. To access silyl ether derivatives of lycorine, initial kinetic monosilylation at the C2 β-allylic hydroxyl group provided direct access to C1 functionalization. Interestingly, the authors report that subsequent treatment of this compound under strongly basic conditions and their desired electrophile resulted not only in the expected C1-alkyl, C2-siloxy-functionalized lycorine, but also in an unexpected C1-siloxy, C2-hydroxyl or C2-alkoxyl-functionalized scaffold, which putatively forms from an intramolecular siloxy transfer reaction, made possible by the elongated Si-O bond compared to alkoxyl substituents, which were not observed to undergo 1,2-migration (Figure 15B). This observation exposes a unique propensity of monosilylated vicinal 1,2-diols to undergo 1,2-silyl transfer reactions, even in incredibly rigid scaffolds such as in lycorine, and with reasonably sterically encumbered silyl protecting groups.

The unexpected silyl migration chemistry (Figure 15B) encountered by the authors was exploited to access both regioisomers of siloxyalkoxylycorine analogs, which were subsequently compared for their biological properties. Throughout six different cell lines (A549, MCF7, T98G, Hs683, SKMEL, B16F10), compounds **15.2**–**15.7**, with at least one triisopropylsilyl (TIPS) on their C-ring and logP > 1, exhibited greater potency on average than analogs lacking a silyl ether, many of which were inactive with GI_50_ > 50 μM. Compound **15.1** (logP = 5.9), possessing a TIPS moiety at C2 and an allyl ether at C1, is equipotent to the parent compound lycorine (logP = −0.8). The biological activity of these analogs seem to be agnostic to the location of the silyl ether, suggesting that the biological activity respecting the C1 and C2 of lycorine is more dependent on cell permeability than filling a hydrophobic pocket. This is further supported by the author’s docking models, which demonstrate how large substituents at either hydroxyl do not perturb direct protein docking. Ultimately, these findings present silyl ethers as a key to tunable hydrolytic stability and hydrophobicity for anti-cancer small molecules whose biological activity is dependent on cell permeability.

### 3.4. Genipin

Genipin, a bioactive iridoid isolated from the fruit extract of *Gardenia jasminoides* and *Genipa americana*, has been extensively studied for its diverse biological activity, from the mitigation of inflammation and ROS generation, to cancer and angiogenesis [187,188,189,190,191]. The versatility of genipin binding, enabled by the susceptibility of its pyran motif to nucleophilic ring opening by a primary amine and subsequent intramolecular ring closing, has manifested in various modes of action that have been unveiled over years of research on this natural product. Its cytotoxic effects are reported in tandem with the inhibition of a myriad of signaling pathways including UCP, STAT, MAPK, and NF-κB, many of which are associated with cancer pathogenesis. These multifaceted biological implications of genipin are therefore attractive areas of investigation from the medicinal chemist’s perspective, providing a broad space to probe structure-activity and structure-function relationship.

**Figure 16:**
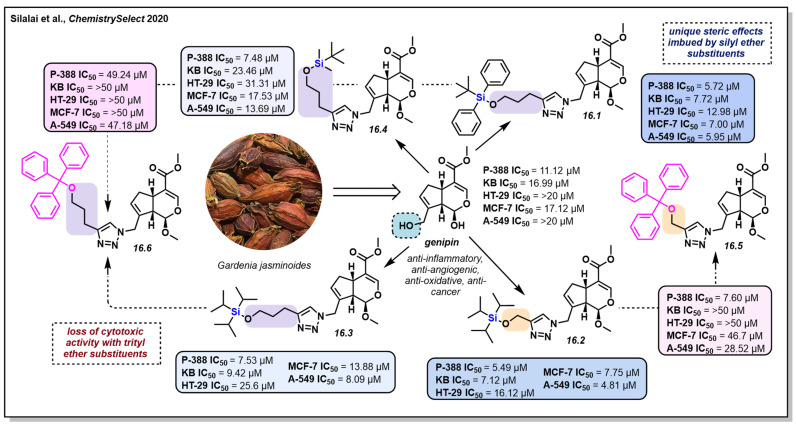
Silyl ether derivatives of genipin exhibit greater antiproliferative activity than their trityl ether counterparts [192]. The various biological functions of natural product genipin render it an intriguing target for structural diversification. Silalai and coworkers recently synthesized a library of genipin analogs with functionalized 1,2,3-triazole rings, including those attached to silyl and trityl ether groups. It was found that while silylated analogs, particularly 16.1, demonstrated improved potency from the parent compound, trityl ether-based compounds of similar steric bulk exhibited little activity across an array of cancer cell lines (photograph of *G. jasminoides* by Fmikas Saisavas, Wikimedia Commons; Published under Creative Commons CC0 1.0).

Seeing the potential in structural diversification of this natural product, Silalai and coworkers explored the functionalization of 1,2,3-triazole rings, motifs known to enhance biological activity in various small molecules drugs, in place of the C10 hydroxyl on genipin [192,193]. Following cell viability assays on 27 triazole-based analogs, it was observed that the construction of different motifs at C4 directly affect the compound’s antiproliferative activity. In particular, silyl ether compounds **16.1**–**16.4** bearing a TBDMS, TBDPS, or TIPS group on a one or three carbon chain linker exhibited increased activity compared to the parent compound across a panel of different cancer cell lines (Figure 16). TBDPS ether **16.1** demonstrated the greatest potency, followed by TIPS compounds **16.2** and **16.3**, and finally TBDMS compound **16.4**, suggesting a sterics-dependent mechanism of binding or interaction with biological targets. However, the replacement of the aforementioned silyl ethers with trityl ether groups of similar size to TBDPS led to a precipitous loss in potency, which corroborates the unique contribution of silicon towards improving the cytotoxic activity of genipin. These findings warrant further inquiry into the potential modifications, via the incorporation of silylated triazoles, of genipin’s biological mechanisms. Furthermore, these silyl ethers likely alter other pharmacokinetic and biophysical properties of genipin that may play a crucial role in their observed antiproliferative activity.

### 3.5. Sesquiterpene Lactones: Tuning an Alkylating Agent

Sesquiterpene lactones (STLs) are a subclass of sesquiterpenoids derived from various *Asteraceae* plants that have traditionally been used to treat inflammation, neurodegeneration, and infections [194,195,196,197,198,199]. However, investigations into the biological activities of STLs have primarily focused on their potent anti-tumorigenic properties, which are attributed to alkylation by the α-methylene-γ-lactone moiety ubiquitous in their structures. Translated into biological contexts, these terpenoids are reported to possess inhibitory effects in cancers that are characterized by overactive NF-κB and/or STAT3 pathways [195,196,197].

**Figure 17:**
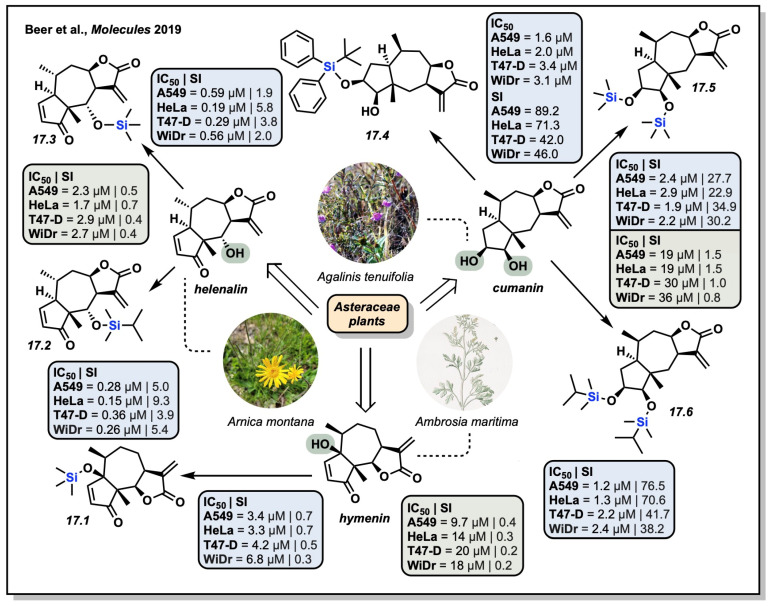
Silyl ether STL derivatives generally exhibit greater antiproliferative activity compared to their respective parent compounds [200]. The sesquiterpene lactone scaffolds offer space for structural diversification via hydroxyfunctionalization. Beer and coworkers recently prepared and evaluated a library of cumanin, helenalin, and hymenin analogs, including a set of silyl ether-based compounds. It was observed that these silylated STLs largely performed better than their parent natural products in a variety of cancer cell lines (photograph of *A. montana*, Elias, Wikimedia Commons, under Creative Commons License CC-BY 4.0; Photographs of *A. maritima* and *A. tenuifolia* obtained from public domain, Wikimedia Commons).

Specifically, the presence of diverse oxyfunctionalization patterns on the B/C ring system of the STL architecture has prompted studies probing the effects of diversification at these positions on cytotoxic activity and selectivity. Recently, Beer and coworkers synthesized twelve analogs of cumanin, helenalin, and hymemin with groups of varying stereoelectronic properties, including esters, alkoxyalkynes, silyl and vinyl ethers, and substituted triazoles [194] (Figure 17). Notably, in vitro results demonstrated that silylated versions of all three natural products significantly outperformed others in the library. TMS helenalin (**17.3**) and dimethylisopropylsilyl (DMPS) helenalin (**17.2**) exhibited nanomolar potency in a diverse array of cancer cell lines, with average GI_50_ values approximately four-fold lower than the parent compound itself. This enhanced antiproliferative activity unique to silyl ether-containing STLs suggests a connection between the increased hydrophobicity and polarizability of silyl ether-containing STLs and their consequential biological activities. While several silyl ether STL analogs exhibited far more potent activity in vitro than their natural counterparts, the analogs of helenalin and hymemin were not observed to significantly amplify the selectivity profiles of the two naturally isolated STLs, warranting further exploration of the mechanisms behind these differences. Nonetheless, the remarkable augmentation in potency imbued specifically by the addition of silyl ethers to these terpenoids supports further exploration of this structure–activity relationship.

### 3.6. Silyl Ethers of Proscillaridin A: Not All Silyl Ethers Improve Potency

Proscillaridin A is a prominent member of the bufadienolide family, a class of cardiac glycosides characterized by a distinctive α-pyrone ring at the C-17 position of the steroidal scaffold [201,202,203,204,205,206]. Traditionally utilized for its positive inotropic effects in the treatment of congestive heart failure [204,205], proscillaridin A has recently gained significant attention for its potent, broad-spectrum antineoplastic activity. Its primary mechanism of action involves the high-affinity inhibition of Na+/K+ ATPase pump [207,208]. By disrupting the electrochemical gradient across the plasma membrane, this natural product triggers a cascade of intracellular events, including the modulation of calcium signaling and the suppression of key oncogenic pathways such as Topoisomerase I/II [209], MAPK, GSK-3β [210], MYC [211], STAT3 [212], HIF-1α [213], and the TRAIL pathway [214], ultimately leading to cell cycle arrest and apoptosis in various malignant cell lines.

The development of silyl ether analogs of proscillaridin A represents a systematic effort to explore the SAR of bufadienolide glycosides. In a study led by our laboratory, two silyl analogs were synthesized among a broader panel of proscillaridin A analogs [215], including proscillaridin siloxy acetonide and bis-siloxy proscillaridin A. These modifications at the sugar moiety significantly increase the lipophilicity of the scaffold with clogP values of 6.53 and 8.86 respectively compared to the natural product with a logP of 2.73. Interestingly, the synthesis of the bis-siloxy derivative demonstrated a threshold for silylation, where even under conditions of high molar excesses of chlorosilane, tris-silylation was not observed on this scaffold, likely due to substantial steric hindrance around the remaining hydroxyl groups of the glycan ring.

**Figure 18:**
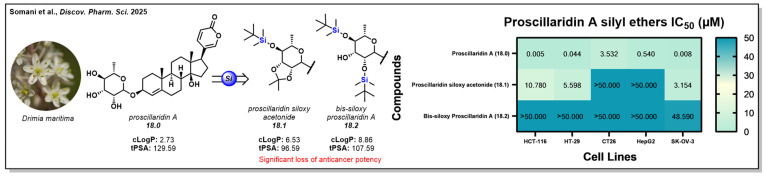
Silyl ethers of Proscillaridin A. Silylated analogs of Proscillaridin A do not display improvements in anticancer potency, suggesting that the addition of lipophilic silyl groups is detrimental to the molecule’s function [213] (photograph of *D. maratima* by Lies Van Rompaey, Wikimedia commons, under Creative Commons License CC-BY 2.0).

In vitro antiproliferative assays across multiple cancer cell lines, including HCT-116 and HT-29 (colorectal) and SK-OV-3 (ovarian), revealed a clear trend of diminished potency following silylation. For example, while the parent proscillaridin A typically exhibits nanomolar potency (IC_50_ = 5 nM), the siloxy acetonide shifted to the low micromolar range (IC_50_ = 10.73 μM), and the bis-siloxy analog was largely inactive (IC_50_ > 50 μM) in HCT-116 colorectal cancer cells (Figure 18). This marked inverse correlation between the addition of silyl groups and biological efficacy suggests that the introduction of large, lipophilic silyl groups onto the rhamnose moiety is detrimental to the molecule’s pharmacophore. This loss of activity was consistently observed across a broad panel of reporter cell lines, specifically those probing the NF-κB, JAK/STAT (STAT1, STAT3, STAT5), and Wnt1/β-catenin signaling pathways. Ultimately, these results demonstrate that while increasing lipophilicity is a common medicinal chemistry strategy, the significantly elevated clogP values (6.53–8.86) imbued by single or multiple silyl ethers do not inherently improve the profile of otherwise potent natural products. Instead, the biological utility of such modifications remains strictly context-dependent, dictated by the specific steric requirements of the target binding pocket and the underlying mechanism of action.

### 3.7. Blue Fluorescent Siloxytecans: Silyl Ethers Imbue Blue Fluorescence for Cellular Imaging

A complementary strategy for incorporating silyl ether motifs was demonstrated in work from our laboratory through the development of fluorescent 10-siloxy analogs of SN-38 for real-time cellular uptake studies [216]. Camptothecin and its derivatives are well-established inhibitors of topoisomerase I (vide supra) exerting cytotoxic effects through the stabilization of the topo I/DNA cleavage complex [95,96,97,217]. Among these analogs, SN-38, (**19.1**) the active metabolite of Irinotecan, displays 100–1000 times greater potency relative to Irinotecan across a wide range of tumor cell lines including those from colorectal, small cell lung, lymphoma, breast, esophageal and uterine cancers [218,219].

**Figure 19:**
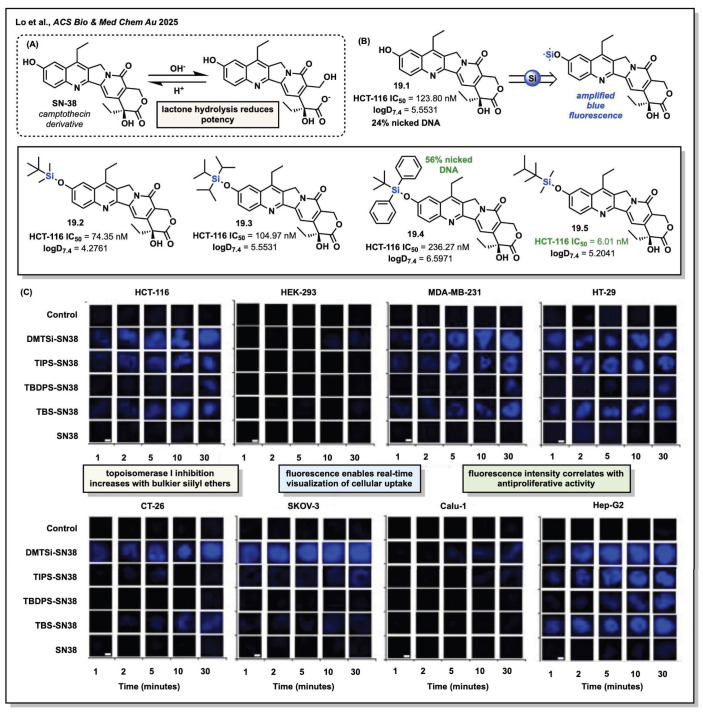
Figure showing 10-siloxytecan derivatives of SN-38 imbue amplified blue fluorescence while maintaining potent antiproliferative activity [216]. (**A**) The pH-dependent hydrolysis of SN-38 from the E-ring lactone to the corresponding carboxylate leads to reduced potency. (**B**) The installation of 10-siloxytecans leads to amplified blue fluorescence in **19.2**, **19.3**, **19.4** and **19.5**, while biological evaluations in HCT-116 indicate little-to-no loss in cytotoxicity. In topoisomerase I/DNA relaxation assays, 19.4, the compound with the greatest logD7.4, displayed 56% nicked DNA in comparison to SN-38 with 24% nicked DNA. (**C**) The effects of the blue fluorescence displayed by these compounds shown in a fluorescence cellular uptake assay over time (fluorescent imaging reproduced with permission from [214], © American Chemical Society 2026).

Despite these advantages, the clinical utility of SN-38 is limited by unfavorable physicochemical and pharmacokinetic properties. The compound exhibits poor aqueous solubility despite its hydrophobicity and is susceptible to pH-dependent lactone hydrolysis into the corresponding carboxylate, a metabolite that is associated with reduced potency [220] (Figure 19A). Additionally, the compound exhibits rapid systematic clearance (CL = 10.2 ± 6 h), partially attributed to UDP-glucuronyltransferase mediated glucoronidation at the C-10 position.

To address these limitations, we prepared a series of 10-siloxy derivatives of SN-38 bearing silyl substituents of increasing steric demand including dimethylthexylsilyl (DMT, compound **19.5**), triisopropylsilyl (TIPS, compound **19.3**), *tert*-butyldiphenylsilyl (TBDPS, compound **19.4**), and *tert*-butyldimethylsilyl (TBS, compound **19.2**). Remarkably, these analogs exhibited intrinsic blue fluorescence with a mean Stokes shift of 45.1 nm, likely due to the electronic perturbation of the electron-donating silyl ethers to the phenol substituent. Biological evaluation across multiple mammalian cell lines, including HCT-116, HT-29, MDA-MB-231, SKOV-3, CT-26, and Calu-1, demonstrated that these siloxytecans retain potent, dose-dependent antiproliferative activity comparable to that of camptothecin and SN-38 (Figure 19). Leveraging this unique fluorescence, these compounds enabled real-time visualization and quantification of cellular uptake. Uptake kinetics were both compound- and cell line-dependent, with maximal intracellular fluorescence detected within 10–30 min of initial exposure. Notably, trends in real-time fluorescence intensity and uptake rates correlated qualitatively with antiproliferative activity across cell lines (Figure 19C).

Topoisomerase I DNA relaxation assays provided additional insight into the functional consequences of silyl ether incorporation on the library of siloxytecans. A general trend of increasing topoisomerase I inhibition with larger alkyl steric demand was observed, with DMT-SN-38 (compound **19.5**) exhibiting the greatest topoisomerase inhibition at 100 μM, with 56% nicked DNA in comparison to SN-38 treatment resulting in 22% nicked DNA at the same concentration (Figure 19B). Collectively, these results demonstrate that the installation of siloxy groups on the E-ring C10 position is a powerful way to perturb both the optical properties and biological behavior of camptothecin derivatives. In addition to retaining potent cytotoxic activities, the unique blue fluorescence exhibited by these compounds enable real-time tracking of cellular uptake, highlighting the potential of siloxy-functionalized SN-38 derivatives as both therapeutic leads and as tools for investigating the cellular uptake of these molecules.

### 3.8. Silyl Ethers as Prodrugs: Decitabine and Its Silyl Ether Analogs of Nucleosides

**Figure 20:**
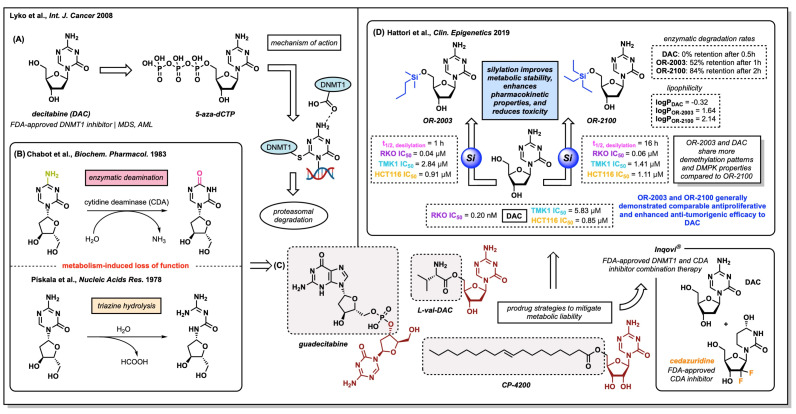
Silylated prodrug analogs of nucleoside derivative decitabine demonstrate complex antiproliferative, physicochemical, and pharmacokinetic trends. (**A**) Upon cellular uptake, decitabine (DAC) is phosphorylated and incorporated into DNA in place of cytosine, wherein it covalently inhibits DNMT1 function and facilitates ubiquitination/proteasomal degradation of the enzyme [221]. (**B**) DAC is vulnerable to metabolic liabilities, namely CDA-mediated deamination and hydrolysis of the triazine scaffold [219,231]. (**C**) Various DAC and azacitidine (AZA) analogs have previously been synthesized as prodrugs to ameliorate losses of potency induced by the aforementioned routes of metabolism. Additionally, combination drug strategies such as Inqovi^®^ have garnered attention as a means to enhance metabolic stability. (**D**) Hattori and coworkers recently explored silylation of DAC as an alternative toward decitabine prodrugs. OR-2100 and OR-2003 emerged as lead analogs, demonstrating unique resemblances to or disparities from DAC in cell-free and biological assays [156].

Abnormal hypermethylation of CpG islands induces the silencing of tumor suppressor genes (TSGs) critical in regulating cellular function, opening the gateway for uncontrolled cell proliferation. Given its role in inhibiting the transcription of these natural regulators through cytosine methylation, DNA methyltransferase 1 (DNMT1) has received particular attention as a promising target for the treatment of various cancers. The design of small molecule inhibitors capable of inducing passive demethylation has been a prominent strategy in this space, dating back to the first syntheses of nucleoside analogs 5-azacytidine and 5-aza-2′-deoxycytidine in the 1960s [221,222]. Extensive studies on their anticancer properties led to their eventual FDA approval and commercial availability as epigenetic drugs, azacitidine (AZA) and decitabine (DAC) respectively, for the treatments of acute myeloid leukemia (AML) and myelodysplastic syndrome (MLS). Both compounds demonstrate remarkable hypomethylating and antiproliferative activity, particularly the latter deoxyribonucleoside, owing to its putative substitution for cytosine during DNA replication. Once bound to DNMT1, the triazine ring of DAC prevents enzyme release that otherwise occurs through β-elimination at the C5 of cytosine, resulting in DNMT1 degradation mediated by the ubiquitin–proteasome pathway and reexpression of previously silenced TSGs [223,224] (Figure 20A).

While highly potent, the efficacies of AZA and DAC are limited by their poor hydrolytic stability and susceptibility to rapid enzymatic degradation by cytidine deaminase (CDA) [221] (Figure 20B). Efforts to improve the longevity and oral bioavailability of these compounds have led to findings that cleavable functionalities at the 3′ or 5′ hydroxyls vastly improve pharmacokinetic properties, as seen with the developments of the dinucleotide guadecitabine (SGI-110) [225], amino acid ester prodrug L-val-DAC [226], and lipidated azacitidine CP-4200 [227] (Figure 20C). Importantly, despite the addition of such bulky groups, these compounds were observed to maintain and, in some cases, improve DNMT1-inhibitory function, hypomethylation, and antitumor activity relative to their parent compounds. Structure diversification has also directed the investigation of other therapeutic strategies to mitigate metabolic liability, one such example being Inqovi^®^, which was approved by the FDA in 2020 as an orally available drug combination of decitabine and cedazuridine that mitigated enzymatic degradation through the latter CDA inhibitor. Notably, the incorporation of two fluorines on cedazuridine plays an important role in its stability under acidic conditions, highlighting the enhanced pharmacological properties which such non-biocanonical elements offer [228] (Figure 20C).

Expanding on heteroatom functionalization, Hattori and coworkers recently probed the prodrug space through a library of 5′-silylated AZA and DAC analogs [156] (Figure 20D). While AZA-based compounds were inactive in an initial DNA demethylation luciferase reporter assay, treatment with certain DAC analogs exhibited comparable activity to decitabine at the optimal 1 μM dose. As such, they were pursued for further evaluation through pharmacokinetic assays. Increased stability toward hydrolysis and enzymatic degradation was observed for analogs bearing greater steric bulk at the silyl ether, albeit excessive spatial demand at that moiety also resulted in poor resistance to CDA-mediated deamination. Consistent with this general sterics trend, dimethyl-*n*-propylsilyl (DMPS) compound OR-2003 displayed a significantly smaller desilylation half-life (t_½_ = 1 h) compared to TES compound OR-2100 (t_½_ = 16 h), and its retention rate in the presence of CDA, though markedly higher than DAC, was also less than that of OR-2100. Notwithstanding differences in enzymatic degradation dynamics, OR-2003 demonstrated greater peak plasma concentration (C_max_) and AUC values that were comparable to DAC, suggesting the complex correlation between the diversity of silyl ethers as protecting groups and the flexibility of DMPK properties.

From the big picture, cell proliferation assays revealed comparable antiproliferative activity between the three compounds, with OR-2003 slightly outperforming OR-2100, and DAC exerting greater cytotoxic effects in certain cell lines. However, a notable exception to this trend was the considerably superior activity of OR-2100 to the other two compounds in DAC-resistant TMK1 human gastric carcinoma cells. Additional disparities between OR-2003 and OR-2100 were revealed by the observation that while both functioned through the primary mode of action of their parent compound, OR-2003 induced greater demethylation of CpG island promoters and tumor-suppressor genes compared to its TES counterpart. OR-2003 and DAC also shared a larger percentage of demethylated genomic regions, relating silyl ether-dependent prodrug metabolism kinetics to intracellular drug behavior. These in vitro results, particularly the resemblance between OR-2003/DAC and the unique growth-inhibitory effects of OR-2100 on TMK1 cells, affirm the tunability of prodrug biological activity concomitant with silyl ether diversification. Furthermore, while OR-2003 demonstrated enhanced reduction of mice tumor volumes, OR-2100 better mitigated off-target toxicity towards white blood cells and liver function. This difference in toxicity, along with an evidently higher logP value and hydrolytic stability for the bulkier DMPS compound, implies a tradeoff between potency and enhanced potential oral bioavailability. Though convoluted, these observations collectively signify that silyl ethers alter prodrug efficacy on multiple bases, from physicochemical and pharmacokinetic properties to their implications for anticancer activity in both in vitro and in vivo models. A similar strategy of siloxyfunctionalization of nucleosides has led to the development of topoII [229] and HDAC inhibitors [230], demonstrating that silylation at C5 of the nucleoside core is a strategic means of structural functionalization of biologically potent nucleoside analogs.

### 3.9. Siloxystearic Acid: Adding a Silyl Ether to a Lipid

**Figure 21:**
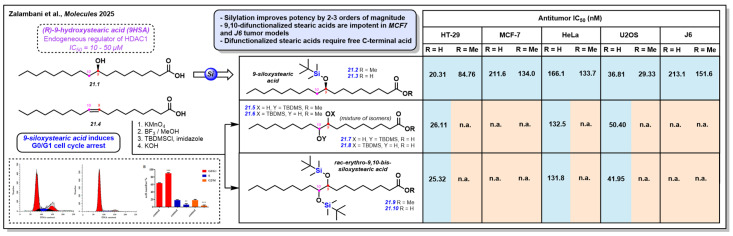
Silyl ethers of (R)-9-hydroxystearic acid greatly increase potency against several cancer cell lines. Moreover, 9-hydroxystearic acid, a known inhibitor of histone deacetylase 1 (HDAC1), exhibits only moderate potency against most cancer cell lines, but silylation at the C9 hydroxyl increases potency by two orders of magnitude or more. Statistical significance was established via *t*-test (** *p* < 0.01, *** *p* <0.001). Flow cytometry data adapted with permission from Zalambani, et al. ([232]), © MDPI 2025).

Given characteristic dysregulation of epigenetic control in multiple cancer models, targeting of enzymes responsible for epigenetic regulation of cell division has demonstrated preclinical and clinical success. R-9-hydroxystearic acid (9-HSA, **21.1**) is a naturally occurring inhibitor of histone deacetylase I (HDAC1), making it an attractive target for synthetic derivatization toward the development of more potent anticancer small molecules [232,233]. Like many aliphatic substrates of HDAC1, 9HSA locks the lysine binding pocket of HDAC1 into an inactive state, thus inhibiting its action in lysine deacetylation on histones and trapping rapidly dividing cells in the G1/G0 phase. In order to evaluate how the silylation of 9HSA and its analogs affects biological potency, a recent report by Zalambani et al. described the chemical synthesis of several silyl ethers of 9HSA (Figure 21) [234]. R-9-(tert-butyldimethylsiloxy)stearic acid (Compound **21.3**) was prepared in two steps directly from 9HSA methyl ester **21.2**. To probe the effect of additional C10 substitution, the authors further prepare a racemic mixture of syn-9,10-dihydroxystearic acid from permanganate oxidation of oleic acid, methylation of the free acid, and mono- or di-silylation of the C9 and C10 hydroxyl functionalities. These siloxy-containing 9HSA analogs were compared against their methyl esters in HT-29, MCF-7, HeLa, U2OS, and J6 tumor cell lines.

Remarkably, both 9-siloxystearic acid and its methyl ester exhibit double- to triple-digit nanomolar potency against all cell lines, with slightly higher potency in its methyl ester, representing an increase in potency three orders of magnitude greater than 6HSA. Consistent with HDAC inhibition, HT-29 cells treated with 9-siloxystearic acid exhibited potent G0/G1 cell cycle arrest and diminished levels of lysine acetylation. However, given the necessity of the free carboxylate terminus in liganding divalent zinc in the HDAC1 binding pocket, the potency of the methyl esters suggest that these compounds may exert their antitumor activity in mechanisms distinct from direct HDAC1 inhibition. By contrast, mono- and bis-silylated erythro racemic mixtures of dihydroxystearic acid were inactive against MCF-7 breast cancer cells and J6 melanoma cell lines, while retaining activity in HT-29, HeLa, and U2OS cell lines, indicating that the methyl ester does not drive selectivity or potency, but rather that C10 functionalization appears to be detriment in the activity of such compounds in MCF-7 and J6 cancer models.

### 3.10. Siloxycyanocinnamic Acids: Filling a Hydrophobic Void Toward MCT1 Inhibition

Hypoxic tumor microenvironments substantially drive cancer cells to adapt to inadequate oxidative phosphorylation by increasing dependence on glycolysis to satisfy abnormally high energetic demands associated with rapid and unchecked proliferation [235,236]. This dependence, in turn, produces excess lactic acid as a byproduct of fermentation, lowering the internal pH of the cytosol and inducing stress upon the cell. Lactate dehydrogenase-mediated regeneration of lactic acid from pyruvic acid, which takes place in the mitochondrial matrix, makes cells in this state dependent on mitochondrial lactate transport.

**Figure 22:**
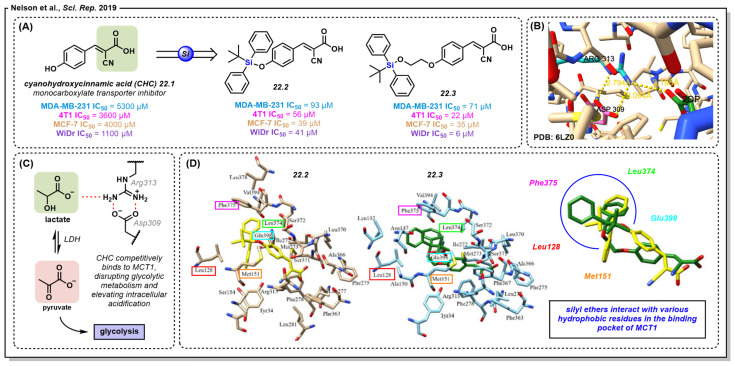
Silyl ether substituents on the C4 hydroxyl of cyanohydroxycinnamic acid interact with hydrophobic residues in the MCT1 transport system, demonstrating an increase of potency up to two orders of magnitude. (**A**) Nelson and coworkers synthesized two tert-butyldiphenyl silyl (TBDPS) ethers with varying alkyl chains that performed significantly better than the parent compound. (**B**,**C**) Lactic acid interacts with Arg313 and Asp309 residues as an endogenous MCT1 substrate. (**D**) Docking studies demonstrate a similar spatial distribution of both compounds bound to the protein with the carboxylic acid moiety interacting with the aforementioned Arg313 and Asp309 residues and the TBDPS silyl ethers interacting with the hydrophobic binding pocket (Binding structures in Panel D reproduced with permission from Nelson, et al. *Sci. Rep.* **2019**, [240], © Springer Nature).

MCT1 is a mitochondrial plasma membrane protein that recognizes the carboxylic acid moiety of lactic acid through electrostatic engagement with an ionically locked Arg313 and Asp309 and facilitates transport into the matrix [125,237]. Targeting this protein has led to the preclinical development of AZD3965 [238,239,240] and other MCT1 inhibitors [241,242], which attenuate cancer cell growth by blocking MCT1-mediated lactate transport. Similarly, the cyanocinnamic acid (CHC) core scaffold has a carboxylic acid moiety that mimics the carboxylate terminus of lactate and binds in close proximity to the same Arg313 residue that recognizes lactic acid (PDB:6LZ0) [243] (Figure 22C). Of these, 4-hydroxycyanocinnamic acid (CHC, 22.1) has been demonstrated to inhibit cancer cell growth through competitive disablement of the carboxylate-binding domain of MCT1 (Figure 22). While CHC demonstrates only modest anticancer potency (IC_50_ = 1–5 mM), Nelson et al. synthesized two analogs of CHC, compounds **22.2** with a TBDPS group and **22.3** with a carbon chain linker between the scaffold and the TBDPS group [242] (Figure 22A). The potency of the silyl ether compounds increased by two orders of magnitude compared to the parent compound in in vitro cell proliferation studies. In cell lines that are more reliant on MCT1, compounds **22.2** and **22.3** demonstrated significantly lower ATP production, lower respiratory capacity, and significantly elevated proton leakage. This exceptional potency and the mitochondrial poison attributes are tentatively credited to the congestion of the MCT1 transport systems mediated by these compounds, whose large silylated functionalities lead to more effective lactate-binding domain blockades (Figure 22D). While MCT1 still recognizes the carboxylic acid moiety, the TBDPS group in both **22.2** and **22.3** occupy a proximal hydrophobic pocket, forging key interactions with M151, L128, P375, L374, and G398, obstructing other substrates like lactic acid from binding to the protein. Furthermore, staining of cells with MTR mitochondria labeling dye after 24 h exposure to 30 μM of compounds **22.2** and **22.3** indicated mitochondrial lysis, further suggesting that this molecule functions as a mitochondrial poison by disabling MCT1 from carrying out its native function.

### 3.11. Silyl Hydrophobic Tags (SiHyT) for Targeted Protein Degradation

**Figure 23:**
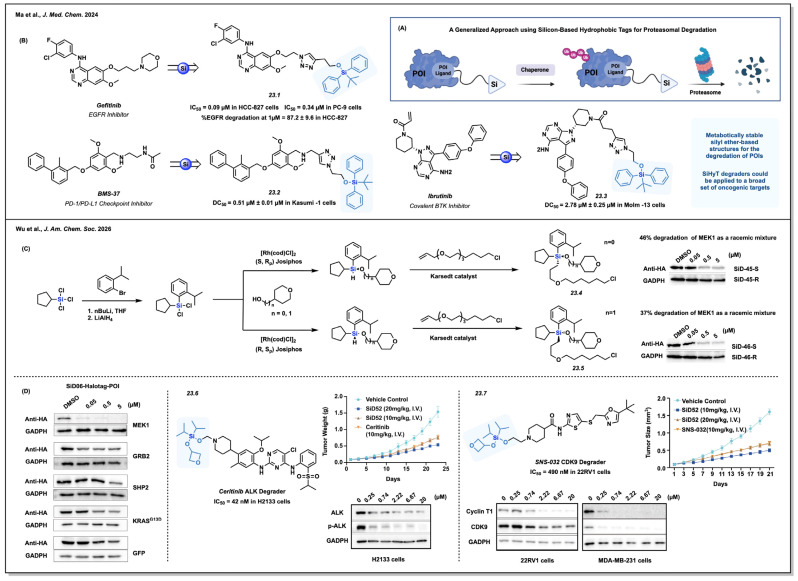
Silicon-based hydrophobic tag degraders (SiHyT) linkers as a generalizable approach for targeted POI degradation. (**A**) Generalizable application of aliphatic silyl ethers as a hydrophobic tag degrader motif. (**B**) Application of *tert*-butyldiphenylsilyl ethers for the preparation of SiHyT tags based on Gefitinib, BMS-37, and Ibrutinib, targeting hydrophobic tag-mediated degradation of EGFR, PD-L1, and BTK, respectively. (**C**) Asymmetric Rh(cod)Cl_2_/JosiePhos-catalyzed functionalization of terminal silanes to chiral difunctionalized silanes. (**D**) Two key examples from Wu et al. demonstrate that a diisopropyl oxetane *bis*-alkoxysilane can be directly applied to ligands that bind to and now drive HyT-mediated degradation of ALK (Compound **23.6**) and CDK9 (Compound **23.7**) and demonstrate potent in vitro and in vivo activity. Graphics reproduced from Ma, et al. *J. Med. Chem.* **2024** ([243]) and Wu, et al. *J. Am. Chem. Soc.* **2026** ([245]) with permission, © American Chemical Society.).

Hydrophobic tagging, an evolving strategy for targeted protein degradation of a protein of interest (POI) through imitation of misfolded protein [244], has historically been highly reliant on protein substrate compatibility, with many effective scaffolds discovered serendipitously rather than through intentional design through the incorporation of adamantane. Serving as a bioisosteric replacement of carbon-based scaffolds, silicon-containing degraders have gained interest due to their enhanced lipophilicity, improved drug-like properties, and diversifiable synthetic accessibility. Ma et al. leveraged the unique hydrophobicity of silyl ethers, deploying this strategy in the preparation of Gefitinib-based hydrophobic degraders targeting EGFR, bearing an aliphatic silyl ether terminus (SiHyT) in lieu of more conventionally explored carbocyclic groups such as adamantyl and menthyl motifs [245]. The authors identify that SiHyT compounds with a tert-butyldiphenylsilyl ether and triazole linker exhibited improved EGFR degradation capacity and enhanced antiproliferative activity over cyclohexyldimethyl silyl ethers, tert-butyldimethylsilyl ethers, triisopropyl silyl ethers, or triethylsilyl ethers (Figure 23A). This lead compound was advanced into in vivo mouse xenograft trials, in which potent tumor reduction and acceptable solubility and PD/PK profiles for oral availability were observed. This initial discovery was further generalized in the development of two advanced candidates targeting PD-L1 and BTK by tethering this triazole-linked *tert*-butyldiphenylsilyl ether to BMS-37 and Ibrutunib, respectively. Both lead compounds demonstrated potent, dose-dependent POI degradation and were found to be orally available (Figure 23B).

Wu and colleagues further elaborated on this strategy, and harnessed modular asymmetric silane functionalization chemistry [246] in the preparation of sixty-four alkoxysilane and bis-alkoxysilane hydrophobic tags, allowing for systematic probing of substituent effects, silicon chirality, and alkoxide stereoelectronics in determining ultimate potency [247]. To elucidate whether degradation was driven by specific substituent effects or by the hydrophobic silicon pharmacophore as a whole, the authors prepared chiral silicon-containing alkoxysilane and *bis*-alkoxysilane motifs with asymmetric Rh(cod)Cl_2_-catalyzed dehydroalkylation of silanes and evaluated both *S*- and *R*- enantiomers (Figure 23C). Given that enantiomers retain identical bulk physicochemical properties yet differ in spatial arrangement, this approach enables differentiation between nonspecific hydrophobic effects responsible for degradation mechanics and stereoselective molecular recognition. Notably, enhancement of moderately performing substrates was observed in the *S*-configuration, while *R*-enantiomer resulted in abated activity, suggesting that the observed biological effects may arise from chiral molecular recognition rather than generalized hydrophobicity. With the identification of an achiral *bis*-isopropyl oxetanyl silane as the lead SiHyT degrader tag, the most potent silicon-degraders in hand, the authors sought to determine whether this strategy of recruitment of degradation machinery might be generalized to a broader subset of diverse protein targets. This was developed into the silyl-tagged analogs of ALK ligand ceretinib ***23.6*** and CDK9 ligand ***23.7*,** which demonstrated potent in vitro activity (IC_50_ = 400 nM in H3122 and IC_50_ = 75 nM in MDA-MB-231) and tumor-suppressive activity in murine xenograft in vivo models (Figure 23D).

The broader implications of this study suggest that protein degradation recruitment may be mediated through defined molecular interactions rather than nonspecific hydrophobic interactions. In turn, silicon-based motifs are proposed to function as unique recognition elements, with chemoproteomic evidence implicating P4HB as a potential interacting partner, possibly through binding engagement within a latent hydrophobic pocket. Collectively, these findings define silicon not merely as a strategy to improve lipophilicity of bioactive drugs, but as a structurally and stereochemically programmable motif capable of directing targeted protein degradation through biological recognition driven mechanisms.

### 3.12. A Rare Example of a Silyl Ester: Mycophenolic Acid, Repurposed for Osteosarcoma

Mycophenolic acid (MPA), a mycotoxin isolated from the *Penicillium brevicompactum*, is a potent inhibitor of IMPDH, an enzymatic target recognized for its essential role in purine synthesis and the production of GTP in all cells. Amongst the two isoforms, IMPDH1 is constitutively expressed in normal leukocytes while IMPDH2 is distinctly upregulated in neoplastic cells [248]. Mycophenolate mofetil, the prodrug derivative of this bioactive metabolite (MPA), has demonstrated therapeutic efficacy towards limiting the proliferation of lymphocytes (T-cell and B-cell), thereby becoming the standard in transplantation medicine and cornerstone in autoimmunotherapy [249]. Subsequent studies have utilized the conjugation between curcumin and MPA to yield a mutual prodrug approach with potent TNF-α inhibitory activity [250]. Beyond its immunosuppressive applications, MPA analogs have also demonstrated activity in other biological contexts within the anticancer space, including hydroxamide derivatives as potent histone deacetylase inhibitors [251], and more recently propargylamine analogs as kinase inhibitors with activity against EGFR, VEGFR-2, and CDK9 [159]. These studies suggest the broader role of mycophenolic acid derivatives as potent anticancer pharmaceutics.

While silyl esters are generally hydrolytically unstable, Silalai and coworkers implemented an array of silicon protecting groups varying in bulk, demonstrating that a triphenylsilyl ester of mycophenolic acid exhibits potent osteosarcoma inhibitory activity. In a broader series of analogs with monosilylated and disilylated ethers and ester attachments varying in steric bulk, aromatic aryl and alkyl substitutions to further probe at the potentially fruitful effects imbued by carbon–silicon bioisosterism, the in vitro evaluation of these analogs across four osteosarcoma cell lines MNNG/HOS, U2OS, 143B, SaOS-2 indicated that organosilicon derivatives (**24.3**–**24.11**) generally exhibited higher activity in comparison to the parent compound MPA and amongst other non-silylated analogs [159].

**Figure 24:**
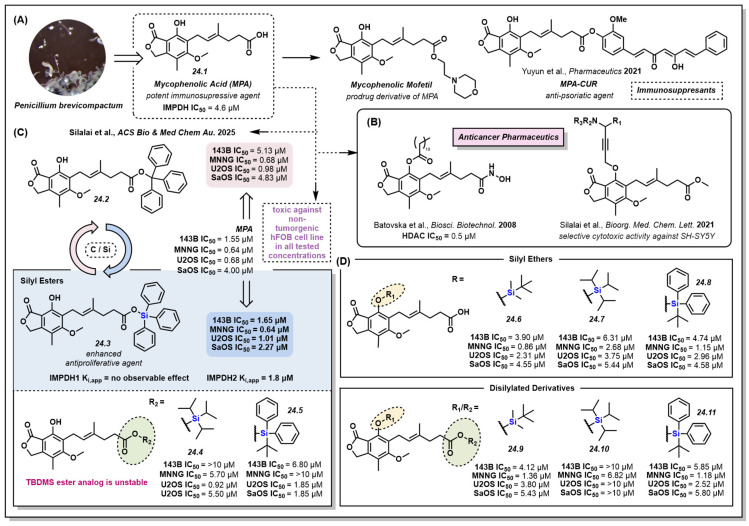
A potent triphenylsilyl ester analog of MPA **24.3** demonstrates enhanced cytotoxicity against osteosarcoma, highlighting a unique application of hydrolytically non-labile silyl esters in medicinal chemistry [159]. (**A**) Mycophenolic acid and derivatives utilized as immunosuppressants [248]. (**B**) Functionalization at the phenol and carboxylic acid pharmacophore has led to the development of several antiproliferative agents [249]. (**C**) Silalai and coworkers demonstrate that organosilicon-containing compounds **24.2**–**24.11** generally exhibit higher activity in 143B, MNNG, USO2, SaOS osteosarcoma cell lines compared to the parent scaffold and other derivatives utilized in the study. (**D**) Bioisostere replacement of a trityl analog with the corresponding silyl ester showed an improvement in biological activity (Photograph of *P. brevicompactum* by Jerry Cooper, Wikimedia commons, under Creative Commons License CC-BY 4.0).

Triphenylsilyl ester compound **24.3** demonstrated comparable potency to its carbon bioisostere compound **24.2** (IC_50_ = 0.68–5.13 µM) across all four osteosarcoma cell lines. While both derivatives substantiated outperformed bioactivity in comparison to other analogs evaluated in the series, the silyl derivative **24.3** (IC_50_ = 0.64–2.27 µM) exhibited enhanced cytotoxic activity, likely due to the advantages of bioisosteric replacement that allowed the bulky silylated side chain to extend further into a hydrophobic pocket modeled in silico within the active site on IMPDH. Remarkably, additional assessment on the cytotoxic selectivity of **24.3** against normal osteoblast cells (hFOB) demonstrated a high tolerability threshold and minimal toxicity (cell viability = 73.34%) at 100 µM, while increasing concentrations of MPA showed detrimental effects (cell viability = 3.38%) accrediting that silyl ester derivatives could potentially be utilized to enhance selectivity. Given the isoform selective but reduced IMPDH inhibitory activity observed in silyl ester **24.3** compared to MPA (Figure 24), it appears that this silyl ester may further attenuate a mechanism of action beyond IMPDH inhibition in cancer cells. The authors propose various cancer-related binding targets of triphenylsilyl ester **24.3**, including CDK2 and VEGFR, wherein the silyl ester occupies a latent hydrophobic pocket as modeled in-silico. Further direct biochemical studies of the target engagement of this compound may afford additional mechanistic information about its activity.

### 3.13. Miscellaneous Examples of Silyl Ether-Containing Bioactive Small Molecules

Beyond the anticancer space covered in this review, others have prepared biologically active silyl ethers of resveratrol for neurodegenerative therapy [252], protein phosphatase inhibitors [253], and human pregnane X receptor agonists [254].

## 4. Siloxanes

Owing to their chemical inertness, siloxanes, characterized by repeat Si-O-Si bonding on tetravalent silicon, have seen a broad variety of use cases in modern materials as lubricants, surfactants, and polymers/polymer precursors. This stability, arising from strong Si-O bonds and flexible bond angles, underpins their widespread use in lubricants, surfactants, and silicone-based polymers. The ring size of discrete cyclic siloxanes is determinant in their stability; while hexamethylcyclotrisiloxane (D3) undergoes ring-opening reactions both in the presence of excessive heat and strong nucleophiles, its eight-membered and ten-membered congeners (octamethylcyclotetrasiloxane, D4; and decamethylcyclopentasiloxane, D5, respectively) are remarkably stable to these and harsher conditions, the former being used as lubricant and thickener in cosmetic products, and the latter being used as a green solvent for dry cleaning.

### Cisobitan: A Cyclic Siloxane for ER-Dependent Cancer Therapy

**Figure 25:**
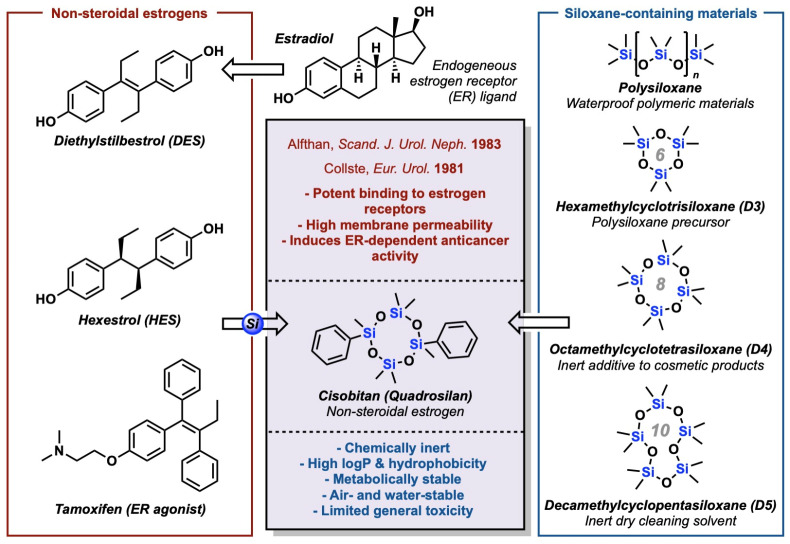
Reapplication of cyclic siloxanes enables the discovery of cisobitan. Cisobitan, bearing remarkable structural similarity to the industrially used octamethylcyclotetrasiloxane (D4), exhibits antigonadotropic activity through its action as a synthetic, non-steroidal estrogen. Given this activity, Cisobitan has been clinically investigated for the treatment of prostate cancers [38,255].

The discovery of non-steroidal estrogen receptor ligands as a strategy for targeting estrogen-receptor-dependent cancers has led to the development of diethylstilbestrol (DES) [255], tamoxifen [256], and hexestrol (HES), which mimic an otherwise planar core structure of the steroid core and competitively bind to the estrogen-binding domain through engagement of two aryl rings spaced with a two-carbon spacer. Cisobitan, which shares the same fundamental eight-membered cyclic siloxane ring found in octamethylcyclotetrasiloxane (D4) but with two phenyl rings placed opposite each other on the siloxane core, shares this biaryl mimicry of estrogen (Figure 25), suggesting a possible spatial mimicry of biaryl estrogen ligands, although the absence of canonical hydrogen-bonding motifs implies a distinct binding mode or indirect endocrine mechanism. In preclinical and clinical studies, cisobitan has demonstrated antigonadotropic properties consistent with ER inhibition and a safety profile of minimal side effects in dosing regimens up to six months consistent with the chemical interness of the eight-membered siloxane ring [38,257,258]. Applied to prostate cancer in comparison to Estradurin^TM^, a polymeric steroidal estrogen receptor antagonist system, cisobitan exhibited minimal benefit, and thus was abandoned from further clinical study. Nonetheless, cisobitan highlights a provocative conceptual direction: the application of cyclic siloxanes that, while exhibiting limited clinical efficacy compared to other ER-based cancer treatments, exhibits remarkable biological compatibility and tolerability. Future efforts that integrate siloxane architectures with well-defined binding motifs may help clarify whether these materials-derived frameworks can be translated into viable bioactive molecules.

## 5. Silicates and Silatranes

**Figure 26:**
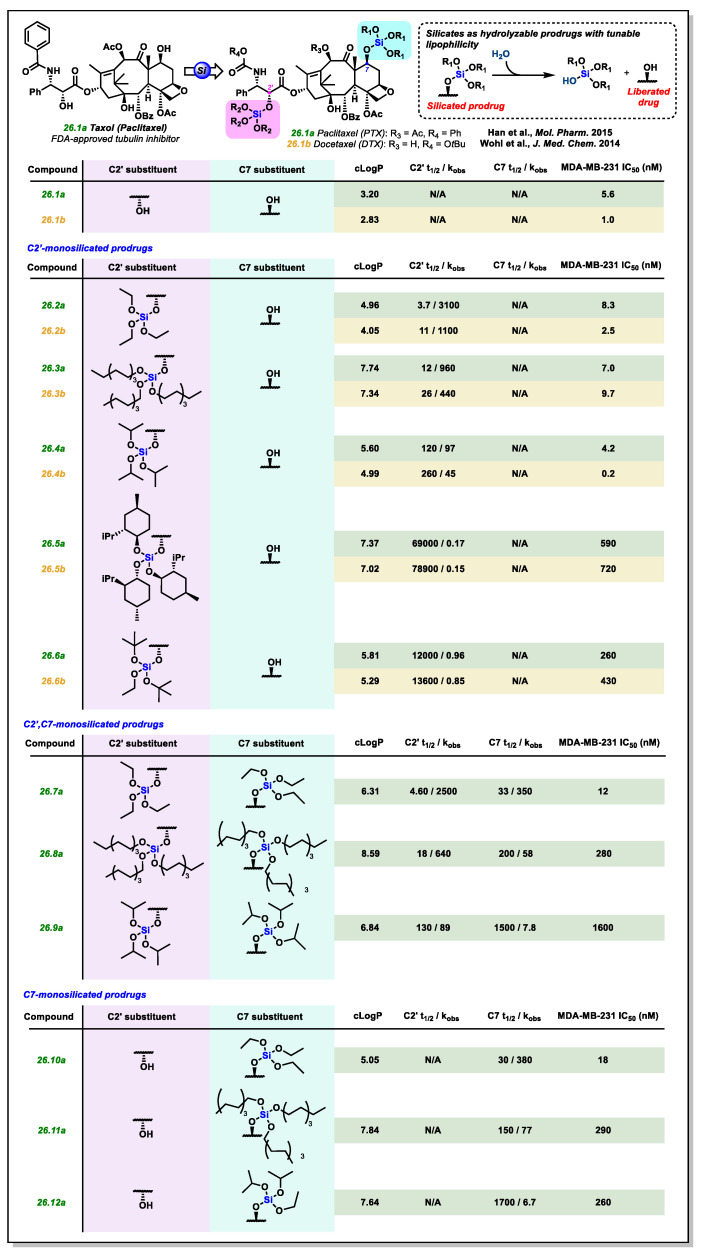
Silicate prodrugs of Paclitaxel (PTX) and Docetaxel (DTX). Generally, this set of compounds exhibits alkyl size-dependent prodrug release kinetics and in vitro potency. Silicate prodrugs at the more sterically demanding C7 position were found to exhibit slower rates of hydrolysis, which corresponds to lower in vitro potency in MDA-MB-231 breast cancer cells [260,261].

While extended silicate materials are among the strongest materials in the world, discrete organosilicates react readily with water to form silicate anions and liberate free alcohols (Figure 26). Linear, aqueous-exposed polysilicates have been similarly demonstrated to hydrolyze in aqueous solution [39,259], leading to the development of a broad variety of biocompatible, hydrolytically degradable biopolymers for the controlled release of therapeutic entities. Recent work from the DeSimone lab, for example, demonstrated this could be applied towards the controlled release of anticancer small molecules such as camptothecin, dasatinib, and gemcitabine, and that the absolute kinetics of this hydrolysis can be carefully controlled by installation of alkyl functionalities on the silicate of varying steric demand [260].

### 5.1. Silicate Prodrugs of Paclitaxel (Taxol) and Docetaxel (Taxotere)

Exploiting this native hydrolytic instability of organosilicates and the tunability of alkyl groups on dictating silicate hydrolysis rates, Hoye and coworkers recently described a new class of paclitaxel (Taxol, **26.1a**) [262] and docetaxel (Taxotere, **26.1b**) [259] prodrugs in which the C2′ and C7 hydroxyl groups of this antimitotic agent were selectively functionalized with a systematic library of linear and branched alkyl silicate functionalities, including triethyl silicate, isopropyl silicate, *n*-octyl silicate, menthyl silicate, and bis-(*tert*)-butylethylsilicates [260,261]. Expectedly, the introduction of silicates at either C2′ and/or C7 significantly increased the lipophilic profile of the prodrugs reported (cLogP = 3.20 and 2.83 for paclitaxel and docetaxel, respectively; in comparison to silicate prodrugs with cLogP > 4.0) with a linear increase in cLogP with increasing aliphatic nature of the trialkylsilicate component. Consistent with known reactivity patterns between the less-hindered C2′ hydroxyl compared to the C7 hydroxyl, hydrolysis rates of analogous trialkylsilicates occurred nearly an order of magnitude slower at the C7 position. Limited differences in C2′-silicate hydrolysis rates were observed between taxol (PTX) and docetaxel (DTX) parent compounds.

In an in vitro cell viability assay, PTX and DTX silicate prodrugs demonstrated differences in antiproliferative IC_50_ values that range from being very similar to their parent compounds (PTX and DTX = 5.60 and 1.0 nM, respectively) to those with IC_50_ values several orders of magnitude higher. Across all compounds, those bearing larger and more hydrolytically stable silicates also experienced higher in vitro IC_50_ values than those with smaller and more hydrolytically reactive silicates (IC_50_ for C2′ triethylsilicates of PTX and DTX = 8.3 and 2.5 nM, respectively, while that of C2′ isopropyl silicates = 4.2 and 0.2 nM, respectively; and that of C2′ trioctyl silicates = 7.0 and 9.7 nM, respectively). By contrast, the triethyl silicate prodrug at C7 of PTX was the only reasonably tolerated compound with silication at that position (IC_50_ = 18 nM) while installation of larger functionalities at C7 including trioctyl and triisopropyl silicates led to a precipitous loss of potency (IC_50_ = 290, 260 nM, respectively), which is concomitant with their remarkable hydrolytic stability (t_½_ = 150–1700 min) indicating that hydrolysis kinetics is a main determinant of prodrug efficacy. Consistent with these observations, highly sterically encumbered silicates such as the di-(*tert*-butyl)ethylsilicate at C2′ (**26.6a** and **26.6b**) exhibit remarkably sluggish hydrolysis (t_½_ = 12,000 and 13,600 min., respectively) and near complete loss of potency in MDA-MB-231 in vitro models (IC_50_ = 260 and 430 nM, respectively). This fundamental hydrolytic stability of trialkyl silicates on either the PTX or DTX core scaffold is highly dependent on the steric demand of the alkyl silicate groups as well as substrate-defined steric environments.

### 5.2. Silatranes: Stability Through a Dative N → Si Bond

Silatranes, a class of tripodal bicyclic aza-siloxanes, represent a scaffold containing a transannular, intramolecular dative bond between nitrogen and silicon, and poses a unique case where silicon is pentacoordinate [263]. Canonically utilized as surface modification agents for the formation of self-assembled monolayers on inorganic material surfaces, its wide applicability has extended into the development of pharmaceutics primarily in the antimicrobial and anticancer space. Owing to the inherent hydrolytic and thermal stability imparted by silatranes, the moiety has nonetheless become a relevant strategy to mitigate poor aqueous solubility and increase bioavailability of potent drugs (bioactive molecules). Singh and coworkers employ this scaffold as a broader series of anthracene based triazolyl silatranes and their silicate counterparts with potential antioxidant and anticancer activity modeled in silico on mutant p53 protein [264].

**Figure 27:**
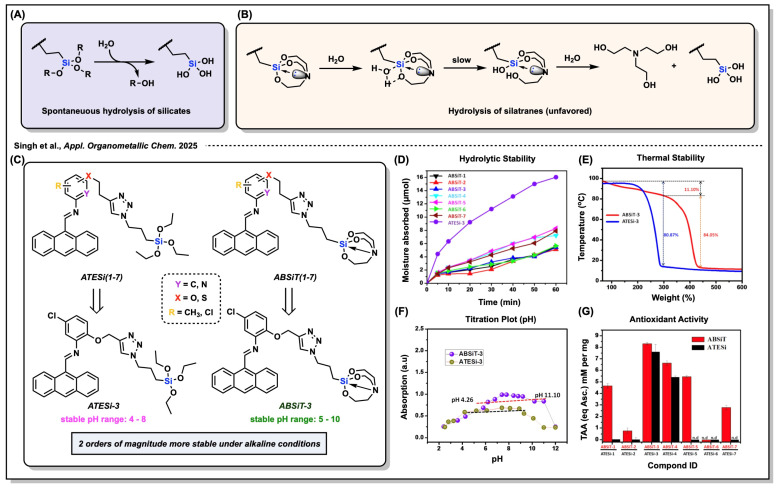
A direct comparison between bioactive schiff-base anthracene derivatives reveals that the pentacoordinate silatrane architecture provides significantly higher hydrolytic stability, thermal resistance, and tolerance towards a broader alkaline range than their silicate counterparts [264]. (**A**) Passive hydrolysis of silicates. (**B**) Hydrolytic attack of the silatrane scaffold. (**C**) Singh and coworkers employ a library of anthracene silicate analogs and their corresponding silatranes to further assess physical and biological activity. (**D**) Rate of hydrolysis between ATESi-3 and ABSiT (1–7). (**E**) Comparison of thermal stability between silatrane analog (ABSIT-3) alongside corresponding silane (ATESi-3). (**F**) Titration plot of ABSiT-3 and ATESi-3 (50 mM) at 274 nm. (**G**) Quantification of total antioxidant activity of compounds ATESi (1–7) and ABSiT (1–7) (stability figures reproduced with permission from [259]. © Wiley VCH).

Compared to the most hydrolytically stable analog ATESi-3 reported in an earlier paper by the same authors, ABSiT-3 demonstrated superior stability when tested in a time-dependent hydrolysis study where the silatrane group maintained fully intact whereas the silane derivative was fully hydrolyzed at 24 h through FT-IR analysis. ABSiT analogs (1–7) were more hydrolytically stable (5–8 μmol) than their corresponding silanes (ATESi 1–7), and also more resistant than ABSi-3 which absorbed 16 μmol of moisture. Thermo-gravimetric analysis (TGA) demonstrates that with the open chain silane ATESi-3 compound exhibits decomposition at approximately 200 °C, while the silatrane ABSiT-3 shows no significant weight loss until 300 °C thereby marking a 100 °C increase in thermal stability thresholds. Furthermore, UV-Vis spectroscopic analysis across a pH range of 2.4 to 12.4 showed that while the silicate is restricted to a stability window of pH 4–8, the silatrane analogs remain intact within a broader range of pH 5–10. This difference of two orders of magnitude increase in alkaline tolerability is important towards its application in the diverse chemical environments within biological systems. Utilizing theoretical computation of molecular orbitals using DFT calculations, it was determined that the silatrane derivatives also pose higher energy gaps (HOMO-LUMO) than their silicate counterparts that would suggest a more kinetically stable and less reactive to spontaneous hydrolysis. Additional assessment on antioxidant activity through a colorimetric assay with select analogs indicate that organosilicon derivatives generally possess higher antioxidant activity than their corresponding silicates (Figure 27). The applicability of a hydrolytically stable hypervalent pentacoordinate silatrane suggests future implications for enhancing aqueous solubility of bioactive molecules and improvement of biological stability of such scaffolds. Already, others have reported this strategy applied in the development of antiviral nucleosides and others bioactive motifs [265]. Importantly, this and other reports demonstrate that natively hydrolytically unstable alkylsilicates (RSi(OR)_3_) can be transformed into caged silatranes with remarkable thermal and aqueous stability simply with the addition of a proximal nitrogen lone pair.

## 6. Conclusions

Intriguingly, the limited incorporation of silicon into biological materials stands in stark contrast to its prevalence as the second most abundant element on Earth. However, silicon-containing organic molecules have been strategically deployed for decades by synthetic organic chemists as reactive intermediates, building blocks, and protecting groups towards the chemical synthesis of complex chemical structures. Recent interest in the therapeutic opportunities offered by the incorporation of silicon and other non-biocanonical elements in small molecule design, concomitant with recent advances in synthetic methodology associated with silicon-containing functional groups, has led to several avenues of re-applying this chemical intuition in the optimization of anticancer small molecules.

In this review, we surveyed a selection of small molecule classes wherein the deliberate installation of silanes, silyl ethers, siloxanes, and organosilicates have offered a modular inroad towards the development of more potent, selective, or tolerable anticancer agents. Additionally, we capture a glimpse of the role of fundamental chemical reactivity of organosilicon-containing functional groups in both the synthetic preparation and downstream intended biological consequences of these subtle chemical changes. Among silanes, several examples from the literature have demonstrated that the minor structural differences between alkylsilicon motifs and their carbon-containing analogous can have profound implications in the downstream biological activities where these silanes are strategically placed to form more effective engagement with hydrophobic pockets, replace carbon-based spacer domains with silane linkers with unique torsional properties, or alter the electronic properties of optically active and photochemically active small molecules. Silyl ethers, which have been broadly used as synthetic protecting groups for alcohols, have taken on new biological roles where the broad array of hydrolytic stabilities can be leveraged as either labile prodrug motifs or metabolically stable masks for hydroxyl functionalities. Siloxanes, which are conventionally found in industrial materials but seldom found in bioactive substances, have been explored as a unique and bioorthogonal spacer ring in the preparation of cisobitan, a cyclosiloxane with antigonadotropic activity. Organosilicates, with their tunable hydrolytic lability and potential for controlled release, represent an additional and comparatively underdeveloped class of silicon-based functionalities with emerging relevance in medicinal chemistry as hydrolytically degradable linkers in biomaterials science as well as in the design of silicate-based prodrugs (Table 2).

Collectively, from the first example of silicon-containing therapeutic modalities in the silaplatin class of DNA intercalators to the modern array of alkylsiloxy functional groups that today enable rapid silyl ether SAR, these studies demonstrate that beyond its role in modulating chemical reactivity of synthetic intermediates, organosilicon incorporation represent a versatile and increasingly appreciated platform for small-molecule drug design.

Contemporary developments further underscore this potential, going beyond the use of silicon as a bioisosteric replacement for carbon with altered lipophilicity and metabolic stability profiles. A growing body of work from multiple groups, including our own, has demonstrated that silyl ether functionalization of natural product scaffolds, including genipin, withaferin A, mycophenolic acid, lycorine, andrographolide, SN-38, and proscillaridin A, among others, the direct impact and functional improvements on potency of silylated natural products are highly context-dependent. Additionally, as detailed in this review, the emergence of silicon-containing prodrug strategies that exploit tunable hydrolytic profiles of both alkoxysilanes and silicates have expanded the role of such sila-functionalities for drug delivery.

In addition, increasing recognition of silicon’s stereoelectronic effects in biological environments has enabled more strategic incorporation of silicon-containing motifs in targeted therapeutics, as illustrated by recent reports of KIF18A inhibitors from Accent Therapeutics, SiHyT-tagged silicon-based degraders, and related systems published in the last year. Advances in late-stage silylation, together with emerging catalytic and asymmetric methods for silicon functionalization, are further enabling exploration of chiral silicon substitution across diverse chemical scaffolds.

While challenges remain in predicting and generalizing the biological consequences of silicon substitution, the continued integration of organosilicon chemistry into drug discovery is likely to expand accessible chemical space and uncover new opportunities for therapeutic innovation. As these strategies continue to mature, organosilicon chemistry will continue to grow as a valuable and evolving component of the medicinal chemist’s toolkit, and is poised to play an increasingly important role in shaping the next generation of small-molecule therapeutics.

## Figures and Tables

**Table 1:** Comparison of physiochemical parameters for carbon and silicon, based on 2,2-dimethylpropane (*neo*-pentane) and tetramethylsilane (TMS) as reference compounds.

Element (X)	Carbon	Silicon
X-C Bond length (Å)	1.54	1.85 [2,21]
X-C Bond dissociation energy (kcal/mol)	83–90	76.0 [46,47]
Van der Waals radius (ppm)	170	210 [48,49]
X(CH_3_)_4_ Rotational barrier (kcal/mol)	4.0–4.6	1.40 [44,45]

**Table 2:** Comparison of chemical and biological reactivities of key organosilicon-containing functional groups, their conventional uses in synthetic organic chemistry, and notable biological applications.

Silyl Group	Synthetic Utility	Biological Application
Silane (-SiR_3_)	Electron rich, sometimes in masking carbon nucleophiles	*Tert*-butyl → trimethylsilyl bioisostere Aliphatic spacers
Silyl ether (-OSiR_3_)	Protecting group for alcohols	Small silyl ethers as hydrolysable prodrugs Large silyl ethers as masked-alcohol analogs
Siloxane (-OSiR_2_-OSiR_2_-)	Chemically inert polymer or polymer precursor	Chemically inert silicon-containing ring
Silicate (-OSi(OR)_3_)	Hydrolysis-prone group	Hydrolyzable prodrug

## Data Availability

No new data were created or analyzed in this study. Data sharing is not applicable to this article.

## Abbreviations

The following abbreviations are used in this manuscript:

AAG	alpha 1 acid glycoprotein
ABC	ATP binding cassette
AML	acute myeloid leukemia
APL	acute promyelocytic leukemia
Aq.	aqueous
ATPase	adenosine triphosphatase
AUC	area under curve
AZA	azacitidine
BBB	blood–brain barrier
BCRP	breast cancer resistance protein
BTK	Bruton’s tyrosine kinase
CA-4	combretastatin A-4
Caco-2	Cancer Coli-2
CDA	cytidine deaminase
CHC	cyanocinnamic acid
CHO	Chinese Hamster Ovaries
cLogP	calculated partition coefficient
CNS	central nervous system
CpG	cytosine–phosphate–guanine
CPT	camptothecin
CRABPs	cellular retinoic acid binding proteins
CYP	cytochrome P450
DAC	decitabine
DB-67	7-t-butyldimethylsilyl-10-hydroxycamptothecin
DFT	Density Functional Theory
DMIPS	dimethylisopropylsilyl
DMOS	dimethyloxysilyl
DMPK	Drug Metabolism and Pharmacokinetics
DMPS	dimethylphenylsilyl
DMT	dimethylthexylsilyl
DMVS	dimethylvinylsilyl
DNA	deoxyribonucleic acid
DNMT1	DNA methyltransferase 1
DPM	diphenyl methane
DTX	docetaxel
EC50	half maximal effective concentration
eEF1A	eukaryotic translation elongation factor 1A
EGFR	epidermal growth factor receptor
ER	estrogen receptor
ER	efflux ratio
FDA	Food and Drug Administration
fLuc	firefly luciferase
FTIR	Fourier-transform infrared
GI50	50% growth inhibition
GTP	guanosine triphosphate
HDAC	histone deacetylase
HSA	human serum albumin
IC_50_	half maximal inhibitory concentration
IMPDH	inosine-5’-monophosphate dehydrogenase
KIF18A	Kinesin Family Member 18A
LC50	lethal concentration 50%
LogP	partition coefficient
MCT1	Monocarboxylate Transporter 1
MDPS	methyldiphenylsilyl
MDS	myelodysplastic syndrome
MPA	mycophenolic acid
NCI	National Cancer Institute
PBS	phosphate-buffered saline
PD-L1	programmed death-ligand 1
PDT	photodynamic therapy
PGP	P-glycoprotein
POI	protein of interest
PTX	paclitaxel
RAR	pan-retinoic acid receptor
ROS	reactive oxygen species
SAR	structure–activity relationship
SEAP	secreted embryonic alkaline phosphatase
SiHyT	silyl hydrophobic tag
SN-38	7-ethyl-10-hydroxycamptothecin
STL	sesquiterpene lactone
STS	steroid sulfatase
TBDMS	tert-butyldimethylsilyl
TBDPS	tert-butyldiphenylsilyl
TBS	tributylsilyl
TES	triethylsilyl
TGA	thermo-gravimetric analysis
TGI	total growth inhibition
THS	trihexylsilyl
TIPS	triisopropylsilyl
TMS	trimethylsilyl
TP-53	tumor protein 53
TPI	tubulin polymerization inhibition
TPRS	tripropylsilyl
TPP	tetraphenyl porphyrin
TPPS4	tetraphenyl porphyrin sulfonate
TPS	triphenylsilyl
tPSA	total polar surface area
TSG	tumor suppressor genes
VDR	vitamin D receptor
WA	withaferin A
*α*-HSA	*α*-hydroxystearic acid
9-NC	9-nitrocamptothecin
